# Increased sinusoidal export of drug glucuronides is a compensative mechanism in liver cirrhosis of mice

**DOI:** 10.3389/fphar.2023.1279357

**Published:** 2023-11-20

**Authors:** Rebekka Fendt, Ahmed Ghallab, Maiju Myllys, Ute Hofmann, Reham Hassan, Zaynab Hobloss, Daniela González, Lisa Brackhagen, Rosemarie Marchan, Karolina Edlund, Abdel-Latif Seddek, Noha Abdelmageed, Lars M. Blank, Jan-Frederik Schlender, Christian H. Holland, Jan G. Hengstler, Lars Kuepfer

**Affiliations:** ^1^ Institute for Systems Medicine with Focus on Organ Interaction, University Hospital RWTH Aachen, Aachen, Germany; ^2^ Leibniz Research Centre for Working Environment and Human Factors, Technical University Dortmund, Dortmund, Germany; ^3^ Department of Forensic Medicine and Toxicology, Faculty of Veterinary Medicine, South Valley University, Qena, Egypt; ^4^ Dr. Margarete Fischer-Bosch Institute of Clinical Pharmacology and University of Tübingen, Stuttgart, Germany; ^5^ Department of Pharmacology, Faculty of Veterinary Medicine, Sohag University, Sohag, Egypt; ^6^ Institute of Applied Microbiology—iAMB, Aachen Biology and Biotechnology—ABBt, RWTH Aachen University, Aachen, Germany; ^7^ Pharmacometrics, Research and Development, Pharmaceuticals, Bayer AG, Leverkusen, Germany

**Keywords:** drug metabolism, liver cirrhosis, sinusoidal transport, glucuronides, drug cocktail, PBPK, systems pharmacology, systems medicine

## Abstract

**Rationale:** Liver cirrhosis is known to affect drug pharmacokinetics, but the functional assessment of the underlying pathophysiological alterations in drug metabolism is difficult.

**Methods:** Cirrhosis in mice was induced by repeated treatment with carbon tetrachloride for 12 months. A cocktail of six drugs was administered, and parent compounds as well as phase I and II metabolites were quantified in blood, bile, and urine in a time-dependent manner. Pharmacokinetics were modeled in relation to the altered expression of metabolizing enzymes. In discrepancy with computational predictions, a strong increase of glucuronides in blood was observed in cirrhotic mice compared to vehicle controls.

**Results:** The deviation between experimental findings and computational simulations observed by analyzing different hypotheses could be explained by increased sinusoidal export and corresponded to increased expression of export carriers (*Abcc3* and *Abcc4*). Formation of phase I metabolites and clearance of the parent compounds were surprisingly robust in cirrhosis, although the phase I enzymes critical for the metabolism of the administered drugs in healthy mice, *Cyp1a2* and *Cyp2c29*, were downregulated in cirrhotic livers. RNA-sequencing revealed the upregulation of numerous other phase I metabolizing enzymes which may compensate for the lost CYP isoenzymes. Comparison of genome-wide data of cirrhotic mouse and human liver tissue revealed similar features of expression changes, including increased sinusoidal export and reduced uptake carriers.

**Conclusion:** Liver cirrhosis leads to increased blood concentrations of glucuronides because of increased export from hepatocytes into the sinusoidal blood. Although individual metabolic pathways are massively altered in cirrhosis, the overall clearance of the parent compounds was relatively robust due to compensatory mechanisms.

## 1 Introduction

Liver cirrhosis is characterized by the replacement of functional parenchyma by scar tissue, which distorts the hepatic tissue architecture and eventually impairs liver function. Cirrhosis leads to reduced protein synthesis, impaired ammonia detoxification, portal hypertension, development of ascites, and hepatic encephalopathy. Importantly, cirrhosis also affects drug metabolism and pharmacokinetics (PK) since the expression of drug-metabolizing enzymes and carriers is significantly altered. Therefore, cirrhosis patients may require dose adjustments to ensure treatment efficacy as well as patient safety (Verbeeck, 2008).

In mice, numerous features of human liver cirrhosis can be recapitulated by repeated administration of the hepatotoxic compound carbon tetrachloride (CCl_4_) for long periods, such as 12 months (Liedtke et al., 2013; Delire et al., 2015; Ghallab et al., 2019b). These mice develop massive fibrosis, regenerative nodules, ductular reaction, reduced expression of cytochrome P450 enzymes in the pericentral lobular zone, compromised ammonia metabolism, and, eventually, hepatocellular cancer (Ghallab et al., 2019b). However, how drug metabolism is functionally altered in these mice has yet to be studied. To address this question, mice were treated with CCl_4_ (or vehicle) for 12 months and then they were administered once with a cocktail containing six drugs after 12 months for functional characterization of metabolism and excretion. Parent drugs as well as their phase I and phase II metabolites were analyzed in blood, urine, and bile. Physiologically based pharmacokinetic (PBPK) models were developed for each drug to investigate physiological processes governing the pharmacokinetics. The drug cocktail consisted of caffeine, codeine, midazolam, pravastatin, talinolol, and torsemide and was specifically designed to facilitate the characterization of specific drug-metabolizing enzymes and drug transporters (Kuepfer et al., 2014). Caffeine, codeine, midazolam, and torsemide serve as probe drugs to study the enzyme activity of human CYP1A2, CYP3A4, CYP2D6, and CYP2C9, respectively. Accordingly, pravastatin and talinolol are probe drugs for the transporter activity of OATP1B1 and MDR1, respectively. The homologous mouse genes to CYP1A2, CYP3A4, CYP2D6, OATP1B1, and MDR1 are Cyp1a2, Cyp3a11, Cyp2d22, Slco1b2, and Abcb1, respectively (Martignoni et al., 2006; Hart et al., 2008; Renaud et al., 2011), while the mouse homolog gene for human CYP2C9 is currently unknown (Hart et al., 2008). For the pharmacokinetic simulations, torsemide was modeled to be metabolized by Cyp2C29, which is the mouse homolog of CYP2C19 (Hart et al., 2008) and is primarily expressed in the liver like human CYP2C9. The selected drug cocktail has been successfully applied in several complementary PBPK studies in humans (Krauss et al., 2017; Fendt et al., 2021) and mice (Thiel et al., 2015; Schenk et al., 2017).

We report a strong increase in glucuronides in the blood of cirrhotic mice, caused by the increased activity of the sinusoidal export of hepatocytes. An unexpected result was that despite the massive downregulation of several cytochrome P450 enzymes, phase I metabolism and the area under the curve of most compounds were surprisingly robust due to the compensatory mechanisms in cirrhotic mice.

## 2 Materials and methods

### 2.1 Mice, induction of chronic liver damage by carbon tetrachloride, and sample collection

Male C57 Black 6/N 8–10-week-old mice were used (Janvier Labs, France). The mice were housed under standard environmental conditions with *ad libitum* feeding on a normal rodent diet (Ssniff, Soest, Germany) and with free access to water. To induce chronic liver damage, the mice were treated repeatedly with carbon tetrachloride (CCl_4_, 1 G/kg intraperitoneal) twice per week for 12 months (Ghallab et al., 2019b; Holland et al., 2022). The control mice received the vehicle only (olive oil, application volume 4 mL/kg intraperitoneal). On day 6, after the last CCl_4_/oil injection, blood as well as liver tissue samples were collected, as previously described (Ghallab et al., 2016). All experiments were approved by the local animal protection authorities (application number: 84-02.04.2017. A177).

### 2.2 Histopathology

Hematoxylin and eosin (H&E) as well as Sirius red staining were performed in 5-µm-thick paraformaldehyde-fixed paraffin-embedded liver tissue sections using standard protocols. For Sirius red staining, a commercially available kit was used (Polysciences Europe GmbH, Eppelheim, Germany) (Ghallab et al., 2021).

### 2.3 Immunohistochemistry

Immunohistochemical analysis was performed on 4-µM-thick paraformaldehyde-fixed paraffin-embedded (FFPE) or 5-µM-thick frozen liver tissue sections. The sections were stained with antibodies against CD45 (BD Biosciences, Franklin Lakes, NJ, United States), K19 (Abcam, Cambridge, United Kingdom), Cyp3a (Biotrend, Cologne, Germany), Cyp1a, Cyp2c (a gift from Dr. R. Wolf, Biochemical Research Centre, University of Dundee, Dundee, United Kingdom), Cyp2e1, (Sigma-Aldrich Corp., St. Louis, MO, United States), glutamine synthetase (BD Bioscience, Germany), or FSP1 (Abcam, Cambridge, United Kingdom) (Ghallab et al., 2019b) (Supplementary Table S1). As secondary antibodies, either anti-rabbit IgG (Agilent, Santa Clara, CA, United States), anti-rat IgG (Linaris GmbH, Heidelberg, Germany), or anti-mouse (Sigma-Aldrich Corp., St. Louis, MO, United States) was used. For target signal visualization, the tissues were stained with either 3,30-diaminobenzidine solution (Vector Laboratories, Peterborough, United Kingdom) or AEC + high-sensitivity substrate chromogen (Agilent, Santa Clara, CA, United States). The nuclei were stained with Mayer’s hematoxylin.

### 2.4 Blood chemistry

ALT, AST, and ALP activities as well as albumin concentrations of plasma samples were measured with a 1:1 dilution in PBS using a Piccolo Xpress^®^ chemistry analyzer and General Chemistry 13 panels (Hitado, Möhnesee, Germany).

### 2.5 Bile acid assay

Plasma concentrations of bile acids were analyzed using negative electrospray (ESI) liquid chromatography-tandem mass spectrometry (LC-MS/MS), as previously described (Ghallab et al., 2022).

### 2.6 Drug cocktail application and sample collection

To check the metabolic capacity of the cirrhotic livers, 12-month CCl_4_- or oil-treated mice were challenged intravenously with a bolus of a drug cocktail containing caffeine (5 mg/kg), codeine (2 mg/kg), midazolam (2 mg/kg), pravastatin (20 mg/kg), talinolol (1 mg/kg), and torsemide (2 mg/kg) (Schenk et al., 2017). The cocktail was administered on day 6 after the last CCl_4_ or oil injection. Blood samples were collected from the retro-orbital sinus time-dependently at 15, 30, 60, and 120 min after the injection of the drug cocktail in a time-dependent manner. Two-hour bile samples were collected from another group of mice via a catheter (SAI-infusion, IL, United States) fixed in the extrahepatic bile duct for the first 2 hours after the administration of the cocktail (Ghallab et al., 2019a). Urine samples (24 hours) were collected from the same mice from which the blood was collected by placing them in metabolic cages immediately after administration of the drug cocktail (Gianmoena et al., 2021).

### 2.7 Quantification of drug cocktail and metabolites

Sample workup of plasma samples and determination with LC-MS/MS was performed as described previously (Schenk et al., 2017). Midazolam glucuronide was determined together with codeine and its metabolites using the MRM transition m/z 518.1 → 324.0 at a fragmentor voltage of 140 V and a collision energy of 22 V. Urine samples were diluted 10-fold and bile samples 20-fold with water prior to sample workup.

### 2.8 Measurement of bile flow rate

To measure the rate of bile flow in cirrhotic and control mice, a mouse catheter (SAI-infusion, IL, United States) was placed in the extrahepatic bile duct of 12-month CCl_4_- or oil-treated mice (day 6 after the last injection), and the outflowing bile was collected as previously described (Ghallab et al., 2019a). Bile collected in the first 30 min was excluded from the analysis to allow a stable bile flow. Subsequently, bile outflow was determined for 2 hours, and the volume of bile was normalized to the body weight.

### 2.9 Bioinformatics of transcriptomics data sets

The chronic CCl_4_ mouse model has been published in Holland et al. (2022), and the data were uploaded to the corresponding publicly available Zenodo archive (10.5281/zenodo.7242764). The raw data are also available at Gene Expression Omnibus (GEO) under accession ID GSE167216. The transcriptomics data of the patients with chronic liver disease were obtained from Holland et al. (2022). These data were downloaded from the Zenodo archive (10.5281/zenodo.7242764). For the analysis of human chronic liver disease, patients with fibrosis stage 6, reported in Hoang et al. (2019), were compared to those with stage 0 as a reference.

All bioinformatics-related analyses were performed in R (version 4.2.1). The source code is publicly available on GitHub (https://github.com/christianholland/cirrhosis-metabolism). If not stated otherwise, a mouse gene was considered differentially expressed if |logFC| ≥ 1 and FDR≤0.05. A human gene was classified as differentially expressed with a more relaxed cutoff of |logFC| ≥ log2 (1.5) and FDR≤0.2. The correlation analysis was performed using the log-fold change (logFC) of human and mouse genes. Therefore, it was required that the gene symbols of both species be in the same name space. Hence, we translated the human HGNC symbols to their ortholog MGI symbols using the R/Bioconductor package BioMart (version 2.44.0), which itself queries the Ensembl Archive Release 99. For the case that many HGNC symbols were translated to the same MGI symbol, the logFC of the resulting mouse gene was the arithmetic mean of the logFC of the human genes. The result of the correlation analysis was summarized as the Pearson correlation coefficient *r*.

### 2.10 Determination of CYP and UGT enzyme activity

Liver microsomes were prepared from frozen liver tissues, as described previously (Lang et al., 2001; Wolbold et al., 2003). Microsomal CYP activities were determined as described previously (Feidt et al., 2010) using 15 µg of microsomal protein in a final volume of 100 µL. Glucuronidation activity was determined as described previously (Johanning et al., 2018) using 20 µg of microsomal protein per 100 µL incubation volume. The concentrations of the probe drugs and incubation times are summarized in Supplementary Table S2. After termination of the incubation, the metabolite concentrations were determined by LC-MS/MS. Two technical replicates were measured per sample.

### 2.11 Statistical analysis

The data were analyzed using GraphPad Prism software version 9.4.0. For statistical analysis, independent samples *t*-test, nonparametric Mann–Whitney test, and 2-way ANOVA were used. *p* < 0.05 was considered statistically significant.

### 2.12 Physiologically based pharmacokinetic modeling

All PBPK models were established in PK-Sim (OSPSuite, Version 9.1, www.open-systems-pharmacology.org) (Kuepfer et al., 2016). Model development was based on the specific ADME properties of each drug; i.e., active transport as well as relevant clearance processes were represented in the PBPK models (Supplementary Tables S3–S20). Physicochemical parameters and information on active processes were retrieved from the literature. Enzymatic clearance processes were defined as first-order kinetics and total hepatic clearance processes accounted for reactions with unknown metabolites. Renal clearances were defined as glomerular filtration unless indicated otherwise. Transport processes followed Michaelis–Menten kinetics, and the Km was kept at the default value of 1 μmol/L; only vmax was optimized. Unknown parameter values were optimized by fitting the PBPK models to the plasma and urine PK data from the control mice. Finally, drug doses were chosen according to the composition of the drug cocktail for the simulations. Detailed information about the model structures and the parameter values is presented in Supplementary Tables S3–S20; Supplementary Figure S1, S2. In addition to simulations in healthy control mice, selected model parameters were additionally modified in the specific PBPK models for further analyses (*see below*). To resemble the situation in cirrhotic livers, metabolic activity was reduced to 11% of the value in healthy control mice. Transporter gene expression was scaled analogously according to the fold change observed from RNA-seq data.

## 3 Results

### 3.1 Liver cirrhosis in mice is accompanied by substantial deregulation of drug metabolizing enzymes and bile acid transporters

**FIGURE 1:**
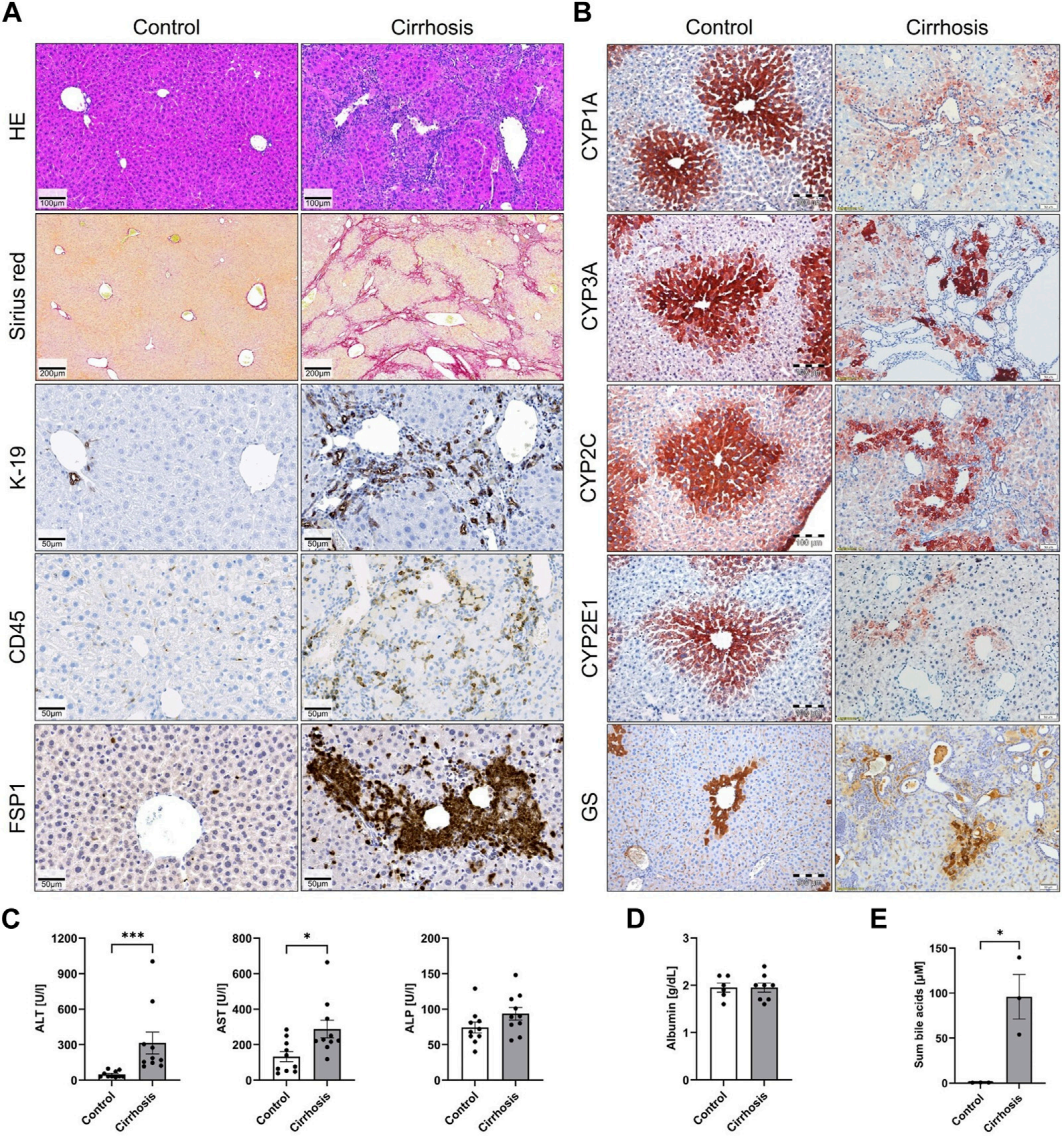
Liver cirrhosis and reduced expression of pericentral cytochrome P450 enzymes after 12 months of CCl_4_ administration to mice. **(A)** Staining with hematoxylin and eosin (H&E) and Sirius red to visualize fibrosis, ductular reaction (K-19), leukocyte infiltration (CD45), and activated macrophages (FSP1). **(B)** Immunostaining of the pericentrally expressed enzymes CYP1A, CYP3A, CYP2C, CYP2E1, and GS. **(C)** and **(D)** Liver enzymes ALT, AST, ALP, as well as albumin and **(E)** serum bile acids in plasma.

Liver cirrhosis in mice was induced by repeated CCl_4_ treatment. After 12 months, severe histological changes were observed in treated mice compared to vehicle (oil) controls (Figure 1A). Wide fibrotic streaks were visualized by Sirius red staining, and a strong ductular reaction was seen, as evidenced by K-19 immunohistochemistry. Moreover, enhanced leukocyte infiltration based on the pan-leukocyte marker CD45 and large areas of FSP1, a marker of a subset of inflammatory macrophages, were observed. A set of enzymes known to be expressed in the pericentral lobular zone, namely, the cytochrome P450 isoenzymes CYP1A*,* CYP3A*,* CYP2C*,* and CYP2E1, as well as the pericentral ammonia detoxifying enzyme glutamine synthetase (GS), were analyzed by immunostaining (Figure 1B). Consistently, the expression of all analyzed pericentral enzymes was strongly reduced after 12 months of repeated CCl_4_ treatment. Plasma ALT and AST and serum bile acid levels increased in the CCl_4_-treated animals, while no significant changes were observed for ALP and albumin (Figures 1C–E).

**FIGURE 2:**
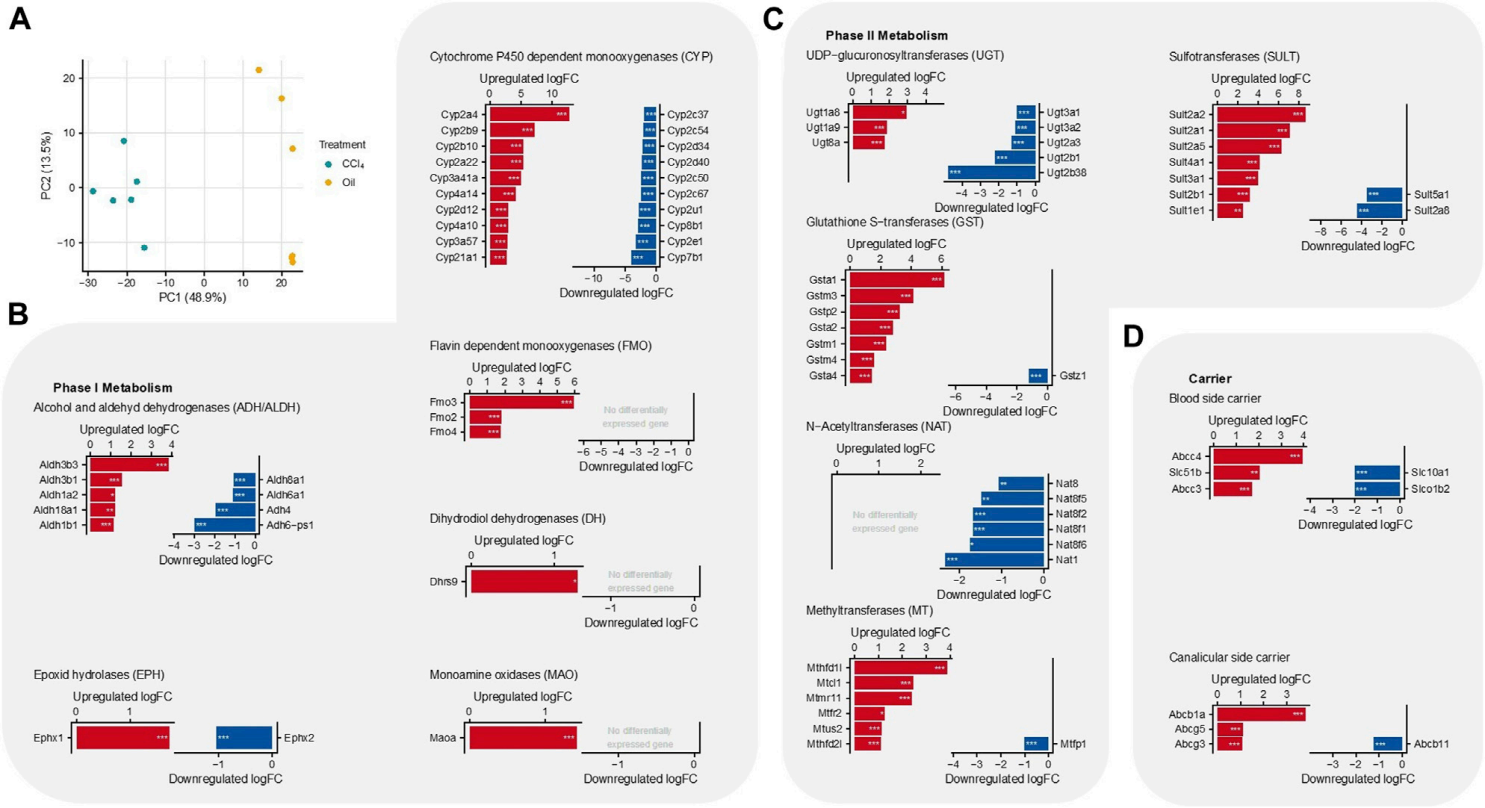
Differential gene expression of drug metabolizing enzymes in cirrhotic liver tissue of mice (12-month treatment with CCl_4_) compared to vehicle (oil) controls. **(A)** Principal component analysis (PCA). **(B)** Phase 1 metabolizing enzymes. **(C)** Phase II metabolizing enzymes. **(D)** Carriers. Six mice were analyzed per group. ***p: 0–0.001; *p*: **0.001–0.01; *0.01–0.1; false discovery rate (fdr) adjusted *p*-values are given.

**FIGURE 3:**
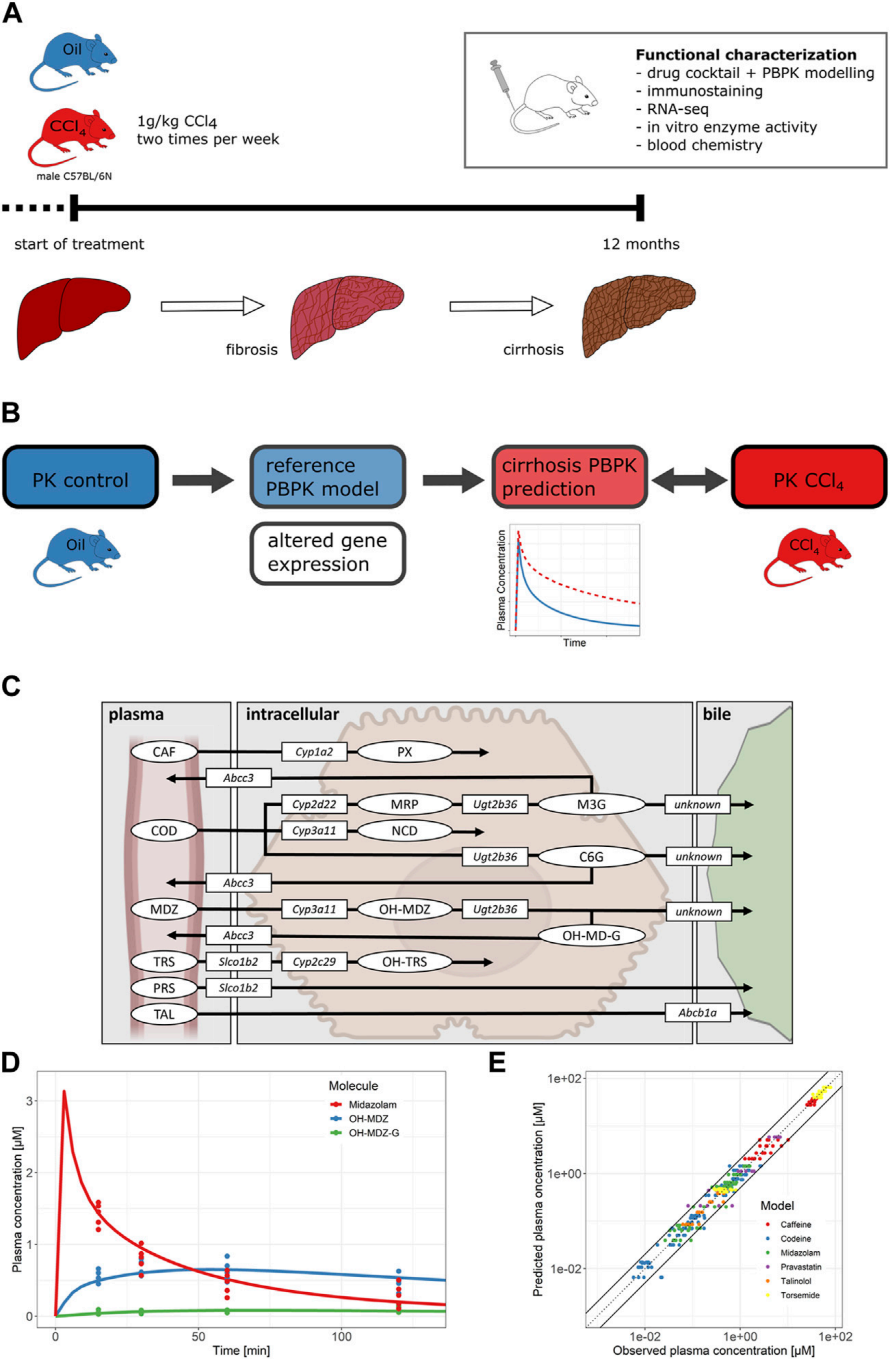
**(A)** Overview of the progression of liver disease in the mouse model and experimental overview. **(B)** PBPK modeling workflow. Different pathophysiological changes were implemented into the reference PBPK models. The PK for mice with cirrhosis was simulated and then compared to the observed data from CCl_4_-treated mice. **(C)** Overview of active processes of the drug cocktail disposition. The figure shows parent drugs (ellipses), metabolites (ellipses), drug-metabolizing enzymes (rectangles), and drug transporters (rectangles) included in the PBPK models. **(D)** Plasma concentrations of midazolam and its metabolites over time. Dots represent the experimental data; solid lines show the reference model simulation. **(E)** Predicted *versus* observed plots of drug and metabolite plasma concentrations for all six PBPK models (metabolites included). Abbreviations: CCl_4_, carbon tetrachloride; CAF, caffeine; PX, paraxanthine; COD, codeine; MRP, morphine; M3G, morphine-3-glucuronide; NCD, norcodeine; C6G, codeine-6-glucuronide; MDZ, midazolam; OH-MDZ, 1-hydroxymidazolam; OH-MDZ-G, 1-hydroxymidazolam glucuronide; TRS, torsemide; OH-TRS, hydroxy torsemide; PRS, pravastatin; TAL, talinolol.

To gain an unbiased overview of the expression of drug-metabolizing enzymes, we re-analyzed a previously published RNA-seq dataset obtained from the liver tissue of six mice either treated with CCl_4_ for 12 months or with the vehicle (4, 14). The two groups of animals clustered distinctly from each other in a PCA analysis (Figure 2A). Analyzing the expression of phase I and II drug metabolizing enzymes and hepatic carriers revealed a complex scenario with significant up- and downregulations in the cirrhotic mice compared to the control mice (Figures 2B–D, Supplementary Material). With respect to the phase I metabolizing enzymes, *Cyp2e1* was among the most downregulated genes, which is plausible because this enzyme metabolically activates CCl_4_ (Hoehme et al., 2010). In addition to a strong downregulation of *Cyp7b1* (Figure 3B), expression of *Cyp7a1,* a key enzyme in bile acid synthesis, was also decreased, although to a smaller degree than that of *Cyp7b1* **(** Supplementary Material). This may represent an adaptive response to increased bile acid blood concentrations in the CCl_4_-treated animals (Jansen et al., 2017). Another striking result among the phase I metabolizing enzymes was the strong upregulation of the flavin-dependent monooxygenase *Fmo3* (Figure 2B). Regarding the phase II metabolizing enzymes, N-acetyltransferases were predominantly downregulated, while glutathione S-transferases, methyltransferases, and sulfotransferases were predominantly upregulated (Figure 2C). Changes in the expression of the hepatic transporters in the cirrhotic mice presented a more straightforward picture. Carriers responsible for the export of bile acids and organic solutes from hepatocytes into the sinusoidal blood, such as *Abcc4*, *Abcc3*, and *Slc51b*, were upregulated, while the sinusoidal uptake carriers *Slc10a1* and *Slco1b2* were downregulated (Figure 2D). Furthermore, the canalicular export carrier *Abcb1a* (multidrug resistance protein) was upregulated, while the bile acid export carrier *Abcb11* (*Bsep*) was moderately but significantly downregulated. Thus, the profiling of drug metabolizing enzymes corresponds to the findings in previous reports (Ghallab et al., 2019a; Ghallab et al., 2019b), and the upregulation of sinusoidal bile acid uptake carriers reflects a situation where hepatocytes protect themselves from bile acid overloading as an adaptation to cholestasis (Jansen et al., 2017).

### 3.2 PBPK models simulate the pharmacokinetics of six drugs with excellent accuracy in healthy mice

Having observed massive up- and downregulations in the expression of phase I and II drug metabolizing enzymes and hepatic carriers in cirrhotic liver, we next studied how drug metabolism is altered in liver cirrhosis. Therefore, a cocktail of six compounds was administered in mice with CCl_4_-induced cirrhosis and non-cirrhotic controls. Parent drugs as well as their phase I and phase II metabolites were analyzed in blood, urine, and bile (Figures 3A–C). As a first step, PBPK models were established and validated in healthy control mice. The PBPK models comprised six parent compounds (caffeine, codeine, midazolam, torsemide, pravastatin, and talinolol), five phase I metabolites (paraxanthine, morphine, norcodeine, 1-hydroxymidazolam, and hydroxy torsemide), and three phase II metabolites (morphine-3-glucuronide, codeine-6-glucuronide, and 1-hydroxymidazolam glucuronide). To this end, CYP-mediated metabolism (Martignoni et al., 2006; Zanger and Schwab, 2013; Hersman and Bumpus, 2014) as well as UGT-mediated glucuronidation (Kurita et al., 2017) were included in the PBPK models. Drug uptake (Slco1b2; (Ogura et al., 2000; Zaher et al., 2008)) and excretion transporters (Abcc3 and Abcc4; (Zelcer et al., 2005; Jarvinen et al., 2021)) at the basolateral (sinusoidal) as well as at the apical (canalicular) hepatocyte membranes were likewise considered (Figure 3C; Supplementary Figures S1, S2, Supplementary Tables S3–S20).

To validate the PBPK models, blood was sampled 15, 30, 60, and 120 min after bolus i.v. administration of the drug cocktail. For example, the simulated (lines) and measured (dots) data of midazolam and its metabolites 1-hydroxymidazolam (OH-MDZ) and 1-hydroxymidazolam glucuronide (OH-MDZ-G) in plasma are shown in Figure 3D, while simulations and data of all other compounds are provided in the Supplementary Figures S1, S2, Supplementary Tables S3–S20. The predicted plasma concentrations for all compounds agree well with the actually measured concentrations since 96.1% of the data points were within the two-fold range of the simulated plasma concentrations (Figure 3E). Moreover, urine was collected for 24 h, and 68.3% of the analyzed data points were within the two-fold range of the simulated concentrations (Supplementary Figure S3).

### 3.3 Glucuronides show an unexpectedly high increase in the blood and urine of cirrhotic mice

**FIGURE 4:**
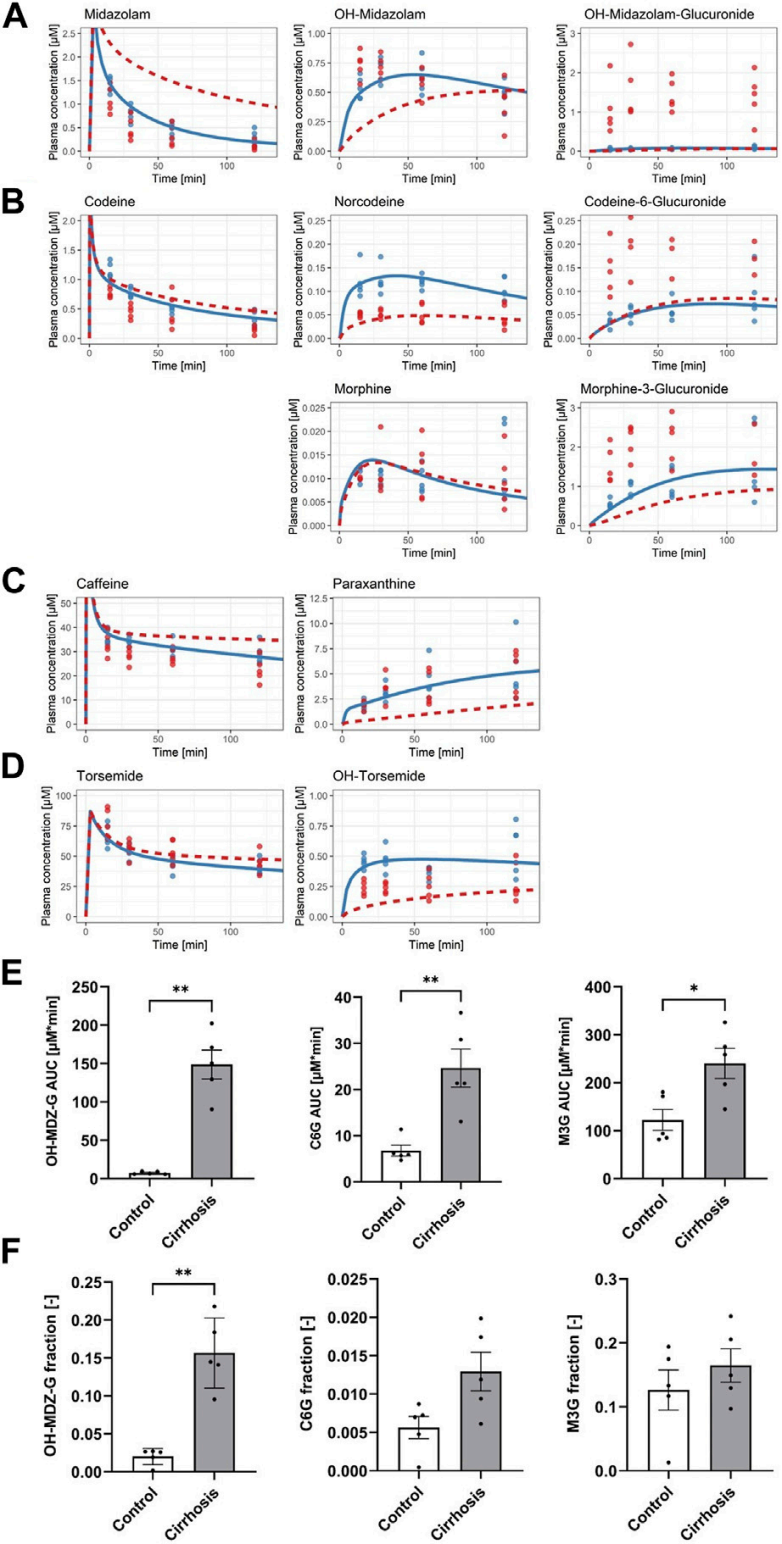
Simulated effect of reduced enzyme activity on drug cocktail disposition. **(A)** Midazolam (parent drug), OH-midazolam (phase I), and OH-midazolam-glucuronide (phase II). **(B)** Codeine (parent drug), norcodeine and morphine (phase I), and codeine-6-glucuronide and morphine-3-glucuronide (phase II). **(C)** Caffeine (parent drug) and paraxanthine (phase I). **(D)** Torsemide (parent drug) and OH-torsemide (phase I). Dots show the observed plasma concentrations; lines show the simulated plasma concentrations. Blue indicates the control group and the reference model simulation. **(E)** Bar plots of plasma AUCs (0–2 h) and **(F)** fractions of the dose excreted in urine (24 h) in control (white) and cirrhotic mice (gray) for OH-MDZ-G (left), C6G (middle), and M3G (right).

Next, model parameters were specifically altered to resemble the situation in cirrhotic livers and the simulation data were compared with experimental data. A key feature of cirrhotic livers of mice after 1 year of treatment with CCl_4_ is the downregulation of CYP1A protein (Figure 1B). Image analysis of sections immunostained for CYP1A showed that the area of the CYP1A expressing hepatocytes was strongly reduced to about 11% of that in healthy animals (quantifications in Ghallab et al. (2019b)). Therefore, we assumed that cytochrome P450 metabolism is generally reduced to that extent and simulated plasma PK of all drugs that undergo phase I metabolism (caffeine, codeine, midazolam, and torsemide) for that case. The result of the simulation was subsequently compared to experimental data obtained from the analysis of blood concentrations of all compounds in cirrhotic mice 15, 30, 60, and 120 min after administration of the drug cocktail (Figures 4A–D). A striking result was that the measured data in cirrhotic mice of all analyzed glucuronides were much higher than the predicted concentrations (Figures 4A, B). This was the case for C6G, the glucuronide of norcodeine; M3G, the glucuronide of morphine; and OH-MDZ-G, the glucuronide of OH-midazolam (Figure 4E). The increased measured concentrations of glucuronides were accompanied by decreased measured concentrations of the parent compounds midazolam and codeine. Comparison with the simulated data suggested that the increased levels of glucuronides may support the excretion of the parent compounds. Out of the 14 plasma AUCs calculated, only those of caffeine and all three glucuronides were increased, while those of norcodeine and OH-torsemide were decreased, and the remaining nine AUCs showed no difference (Supplementary Figure S4).

A well-known consequence of glucuronidation is increased urinary excretion. Therefore, we tested the urinary concentrations of all three glucuronides in cirrhotic mice and in the corresponding time-matched vehicle controls. Indeed, C6G, M3G, and OH-MDZ-G showed higher fractions of dose in the urine of cirrhotic than in control animals (Figure 4F). However, the changes only amount to statistical significance for OH-MDZ-G (*p*-value: 0.008) but not for C6G (*p*-value: 0.056) and M3G (*p*-value: 0.548) due to the complexity of codeine drug metabolism (Figure 4F; Supplementary Figure S5).

### 3.4 Elevated glucuronide blood levels are caused by increased hepatocyte export to sinusoidal blood

**FIGURE 5:**
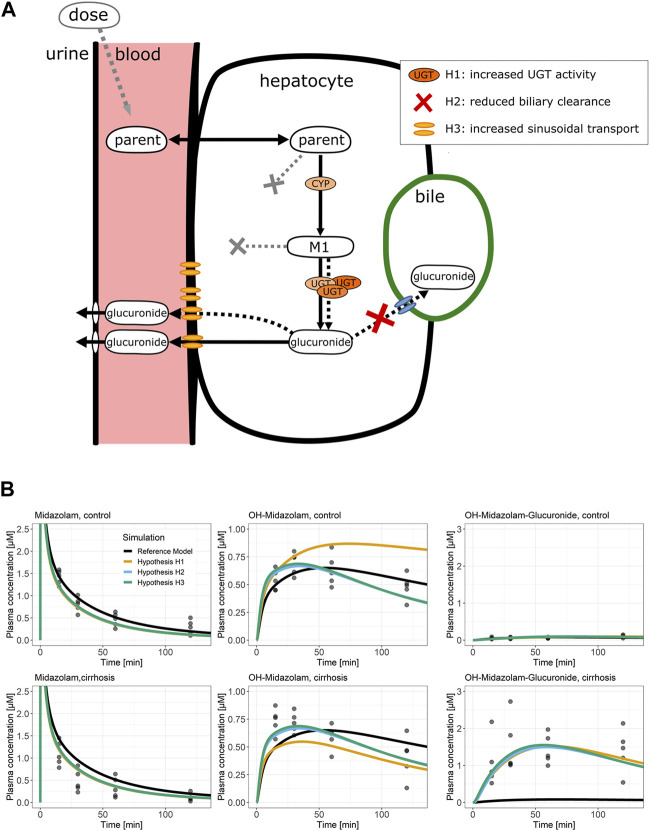
Potential pathomechanisms causing altered glucuronide disposition. **(A)** Schematic representation of processes that could be altered in mice with CCl_4_-induced cirrhosis. Three hypotheses could explain the increased glucuronide concentrations in plasma: increased activity of phase II enzymes (H1), reduced biliary clearance (H2), or increased sinusoidal export (H3). **(B)** Midazolam PBPK simulations of the three hypotheses. Dots display observed plasma concentrations; lines display the PBPK model simulations. The line color specifies the simulated PBPK model fit. The plots in the upper row show the control data and simulations, whereas the plots in the lower row show the cirrhosis data and simulations. Abbreviation: CCl_4_, carbon tetrachloride; M1, phase I metabolite; PBPK, physiologically based pharmacokinetic; OH-MDZ-G, 1-hydroxymidazolam glucuronide.

We next studied which mechanisms may explain the increased concentrations of glucuronides in the blood of cirrhotic animals. We first tested several hypotheses using structural model extensions followed by experimental data (Figure 5A). One possible mechanism is that cirrhotic mice have increased activities of UDP-glucuronosyltransferases (UGTs) (hypothesis H1). A second hypothesis is that there is reduced biliary clearance of glucuronides, which leads to increased export into the sinusoidal blood, although this is not accompanied by increased sinusoidal export transporter expression (hypothesis H2). A third possibility is the increased export of glucuronides into the sinusoidal blood via the basolateral hepatocyte membrane (hypothesis H3). According to hypothesis 3, there is no increased formation of glucuronides in hepatocytes, but sinusoidal export occurs more efficiently. Of course, combinations of all three hypotheses are also possible.

To test these hypotheses by model simulations, the PBPK models of midazolam and codeine were used since these were the only two compounds that form glucuronides. The models were fitted simultaneously for cirrhotic and control mice to the PK data of parent drugs as well as phase I and phase II metabolites. Additionally, either UGT activity (H1), biliary clearance (H2), or the activity of sinusoidal export (H3) was independently optimized (Supplementary Table S21). Interestingly, the model fits demonstrate that all three hypotheses can describe the increased glucuronide blood concentrations in cirrhotic mice (Figure 5B; Supplementary Figures S6–S8).

**FIGURE 6:**
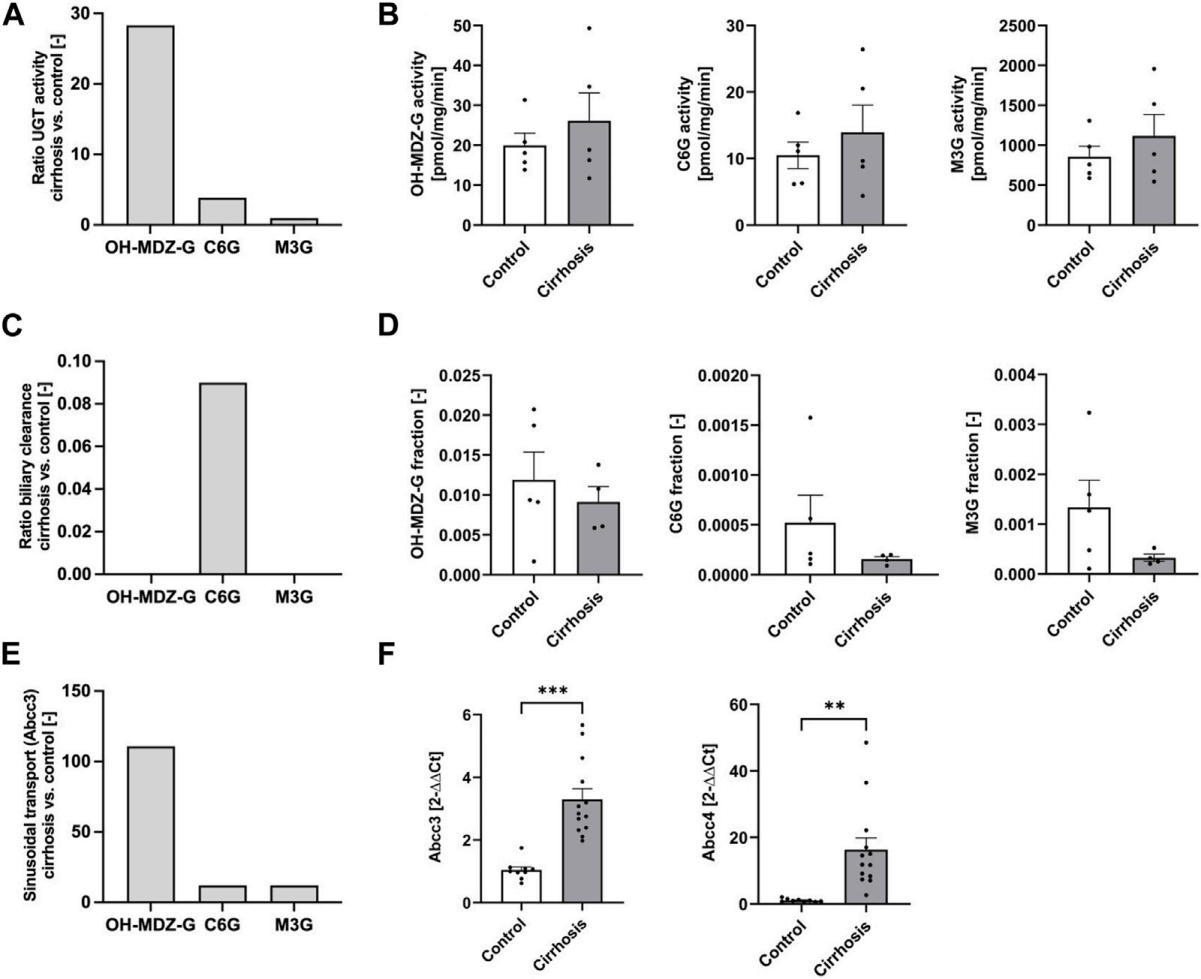
Comparison of model simulations **(A,C,E)** with experimental data **(B,D,F)**. **(A)** Ratio of UGT activity (cirrhosis vs. control) for OH-MDZ-G, C6G, and M3G in hypothesis H1. **(B)** Metabolite formation rates in liver microsomes of M3G, C6G, and OH-MDZ-G. **(C)** Ratio of biliary clearance (cirrhosis vs. control) for OH-MDZ-G, C6G, and M3G in hypothesis H2. **(D)** Fractions of dose excreted in bile of OH-MDZ-G, C6G, and M3G. **(E)** Ratio of increased sinusoidal transport (cirrhosis vs. control) for OH-MDZ-G, C6G, and M3G in hypothesis H3. **(F)** RNA expression of the sinusoidal drug transporters *Abcc3* and *Abcc4*. Bars display fold changes compared to the oil-treated mice. Abbreviation: CCl_4_, carbon tetrachloride; C6G, codeine-6-glucuronide; M3G, morphine-3-glucuronide; OH-MDZ-G, 1-hydroxymidazolam glucuronide. **(A,C,E)** Bars quantify specific parameter results from the computational models H1, H2, and H3 (Figure 5; Fig. Supplementary Tables S22, S23) **(B,D,F)** bars display the mean value and the standard error.

To further examine hypothesis H1, model simulations were compared to experimental data, specifically to activities of UGT in liver microsomes. To further explain the increased plasma concentrations in cirrhotic mice, the model predicted a 28-fold increase of UGT for OH-MDZ-G, a 3.9-fold increase for C6G, and no changes for M3G (Figure 6A; Supplementary Tables S22, S23). However, a comparison with UGT activity in liver microsomes showed that enzyme activities of phase II metabolism were not significantly different between cirrhotic and control mice (Figure 6B). Therefore, hypothesis 1 was disproven by the experimental data.

For hypothesis H2, the simulated biliary excretion rates in the cirrhotic animals were extremely low in the PBPK models (Figure 6C; Supplementary Tables S22, S23). No significant differences between cirrhotic and control animals were obtained for the glucuronide concentrations in the bile (Figure 6D; Supplementary Figure S9). Likewise, no difference could be identified in the flow rates of extrahepatic bile and the expression of canalicular glucuronide transporter *Abcc2* (Supplementary Figure S10). Therefore, the experimental data also disprove hypothesis 2.

For hypothesis H3, the model simulations showed that the sinusoidal export activity of hepatocytes has to be increased by about 110-fold for the midazolam model and by 12-fold for codeine to explain the observed glucuronide blood concentrations in cirrhotic mice (Figure 6E; Supplementary Tables S22, S23). Importantly, RNA-seq of liver tissue showed significantly increased mRNA expression of sinusoidal glucuronide transporters *Abcc3* (log 2 FC = 1.69; *p* = 3.28 E -09) and *Abcc4* (log 2 FC = 3.95; *p* = 9.07 E-19), which are known to mediate excretion of glucuronides from hepatocytes (Zelcer et al., 2005; Jarvinen et al., 2021). qRT-PCR confirmed the elevated transporter expression, more specifically that expression of *Abcc3* mRNA increased about 3-fold and *Abcc4* about 16-fold in the livers of cirrhotic mice (Figure 6F). In conclusion, the experimental data qualitatively agreed with hypothesis 3—that the formation of glucuronides in hepatocytes is not increased, but the sinusoidal export occurs more efficiently, while hypotheses 1 and 2 were rejected.

### 3.5 Overall phase I metabolism in cirrhosis is robust despite the downregulation of pericentral CYPs

As summarized previously, a strong decrease in the expression of phase I metabolizing enzymes in cirrhotic compared to control livers was observed (Figure 1B), in particular CYPs, which are expressed in the pericentral region of the liver lobule. In cirrhotic livers, *Cyp1a2* and *Cyp2c29* showed reduced RNA levels, and immunostaining demonstrated strongly reduced pericentral areas of *Cyp1a*- and *Cyp2e*-positive hepatocytes (Figure 2B). As described previously, we simulated the metabolism of all four drugs which undergo phase I metabolism assuming that cytochrome P450 metabolism, in general, is proportionally reduced to the area of CYP1A-expressing hepatocytes (Figures 4A–D). These simulations predicted the CYP metabolites to be lower in the blood of cirrhotic than in control livers, with the exception of morphine (Figure 4B). The model predictions for the phase I metabolites paraxanthine (Figure 4C), OH-torsemide (Figure 4D), and OH-midazolam (Figure 4A) were not confirmed by the experimental data, which showed no significant difference between the cirrhotic and normal livers; only for norcodeine did the model correctly predict reduced formation in cirrhotic mice, which was experimentally validated (Figure 4B). For the simulations of parent compound concentrations, a strongly (midazolam) or moderately (caffeine, codeine, and torsemide) reduced clearance was obtained for the cirrhotic compared to the control conditions (Figure 4). In addition, this model prediction was not validated by the experimental data. Thus, for several drugs, delayed clearance of parent compounds and reduced formation of phase I metabolites, as suggested by the model in cirrhotic livers, were not validated by the experimental data. A striking example is that of caffeine, which is known to be metabolized to paraxanthine by *Cyp1a2*. In contrast to the expected results, the clearance, and consequently, the AUC of caffeine, was even decreased in cirrhotic mice, suggesting increased metabolism (Supplementary Figure S4).

To address the question of why phase I metabolism is so stable in cirrhotic livers, we performed activity assays using microsomal preparations from cirrhotic and control livers using caffeine, codeine, midazolam, and torsemide as substrates (Supplementary Figure S11, Supplementary Table S2). Liver microsomes contain CYPs and other membrane phase I enzymes and, therefore, are suitable for evaluating the phase I metabolic capacity of hepatocytes. Formation rates of the five phase I marker metabolites in the microsomal assay were measured (Supplementary Figure S11), and the ratio of the mean rates for control and cirrhotic mice was calculated. Interestingly, this ratio of means did not differ significantly between the cirrhotic and control groups (Table 1). Therefore, the analyses show that despite the downregulation of some CYPs, the overall phase I metabolic capacity of microsomal preparations was not reduced in cirrhotic mouse livers for most of the studied drugs. This corresponds to the observation that besides the downregulation of several CYPs, other CYP isoforms and further phase I metabolites were upregulated (Figure 2). Of note, a substantial influence of pathophysiological alterations, such as ascites or changes in hepatic blood flow, which occur in human cirrhotic patients, could be ruled out through additional model simulations (Supplementary Figures S12, S13; Supplementary Tables S24–S26).

**TABLE 1:** Overview of active drug disposition processes, relevant ADME gene, gene expression, and enzyme activity. Ratio of means was calculated from the in vitro CYP activity assays (Supplementary Figure S11).

Drug	Active process	ADME genes			Microsomal activity assay	
			log_2_FC	*p*-value	Ratio of means	*p*-value
Caffeine	Caffeine–> paraxanthine	*Cyp1a2*	−1.69*	4.07E-07	0.93	0.15
Codeine	Codeine–> morphine	*Cyp2d22*	−0.18	0.23	1.32	0.42
	codeine–> norcodeine	*Cyp3a11*	0.30	0.46	0.76	0.15
Midazolam	Midazolam–> OH-MDZ	*Cyp3a11*	0.30	0.46	1.27	0.42
Torsemide	Torsemide–> OH-torsemide	*Cyp2c29°*	−1.92*	1.01E-09	0.86	0.69

log_2_FC: logarithmic fold change to base 2*: significant, °: assumption in the PBPK model, synonymous gene or protein names are listed in brackets.

### 3.6 Changes in transporter gene expression influence renal as well as biliary excretion

The AUCs of the two transporter probe drugs pravastatin and talinolol were found to be unchanged in control and cirrhotic mice (Supplementary Figure S4). This is in agreement with PBPK simulations for reduced *Slco1b2* (pravastatin and torsemide) and *Abcb1* (talinolol) expression, respectively (Figure 2; Supplementary Figure S14). In contrast, the fraction of the dose excreted in urine increased significantly for both pravastatin and torsemide, indicating that impaired sinusoidal uptake by *Slco1b2* is compensated through increased renal excretion in this case (Supplementary Figure S6). Hence, decreased formation of OH-torsemide (Supplementary Figure S4) could also be caused by impaired hepatocellular uptake due to reduced *Slco1b2* expression instead of reduced *Cyp2c29* metabolism. Talinolol, in turn, was significantly increased in the bile of cirrhotic mice (Supplementary Figure S14), which agreed with the increased RNA expression of *Abcb1a* and qualitatively also with the PBPK model prediction (Figure 2; Supplementary Figure S14).

### 3.7 Translational relevance

**FIGURE 7:**
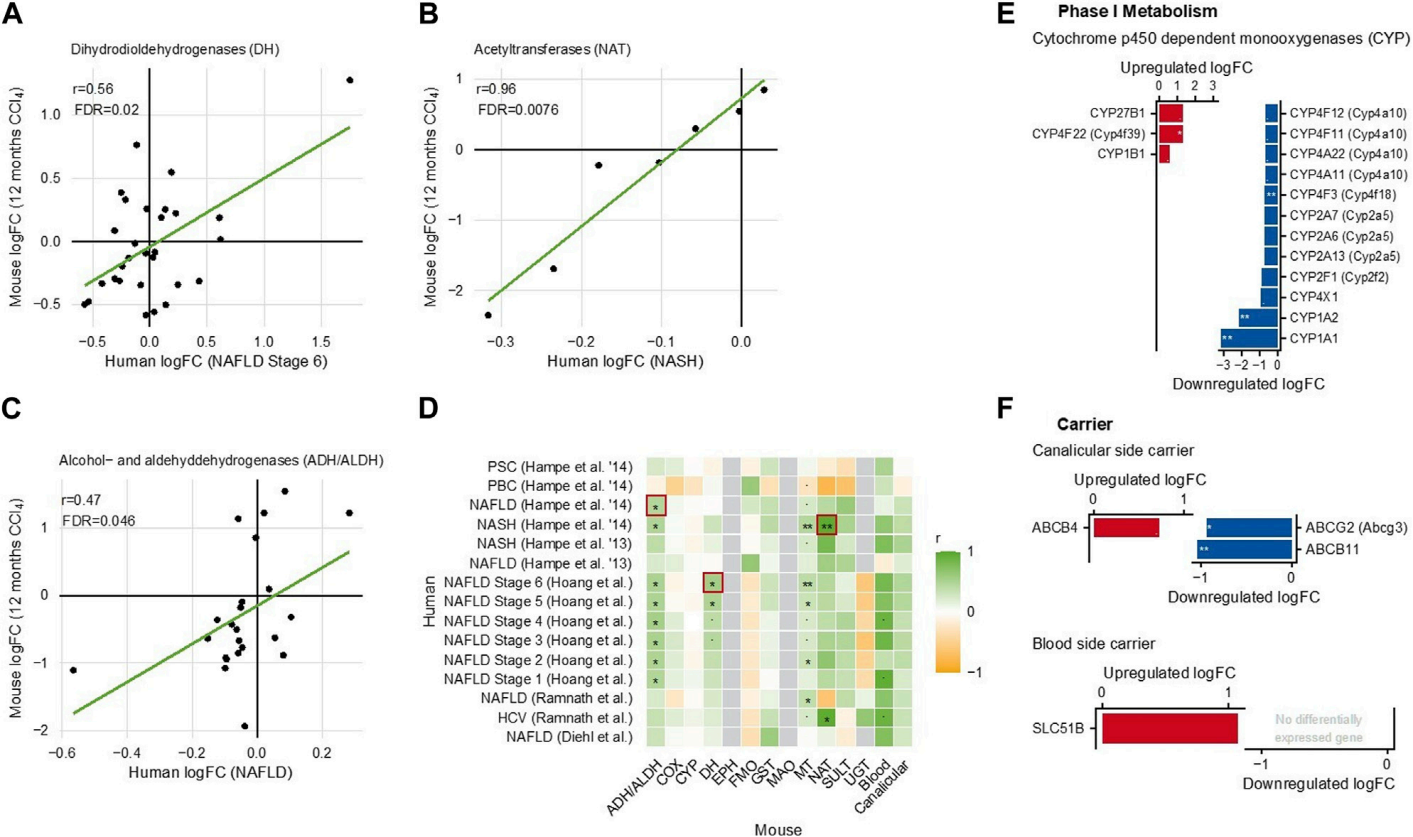
Comparison of gene expression changes in cirrhotic mouse livers and in chronic human liver disease (Holland et al., 2022). **(A)** Dihydrodiol dehydrogenases. **(B)** N-acetyltransferases. **(C)** Alcohol- and aldehyde-dehydrogenases. Each dot in **(A–C)** represents an individual gene. **(D)** Heatmap summarizing all analyzed mouse-human correlations. **(E)** Phase I metabolizing enzymes; and **(F)** carriers differentially expressed in human liver disease.

Finally, an important question is whether the changes in drug metabolism induced in mice by chronic CCl_4_ administration are relevant for humans. To address this, we used a previously published database of publicly available genome-wide datasets of chronic human liver diseases (compared to controls), including primary sclerosing cholangitis (PSC), primary biliary cholangitis (PBC), non-alcoholic fatty liver disease (NAFLD) ranging from stage 1–6, non-alcoholic steatohepatitis (NASH), and hepatitis due to HCV (Holland et al., 2022). Corresponding human and mouse genes were identified, and the correlation of their fold-changes (cirrhosis *versus* control in mice; tissue from patients with chronic liver disease *versus* controls, as described by Holland et al. (2022)) was analyzed (Figure 7).

Overall, the fold changes were smaller in humans than in mice. To give an unbiased overview, all analyzed mouse–human correlations were summarized in a heatmap (Figure 7A). This overview shows—in general—statistically significant mouse–human correlations for ADH/ALDH (Figure 7B), DH (Figure 7C), NAT (Figure 7D), and carriers of the blood and canalicular side of hepatocytes. In contrast, mouse–human correlations were generally low for CYP and UGT (Figure 7A). However, when considering the individual enzymes, some CYPs including CYP1A2 (the major caffeine metabolizing CYP) were also decreased in human chronic liver diseases, similarly to mice (Figure 7E). The human blood side export carrier *SLC51B* was upregulated, suggesting that the principle of increased sinusoidal export may also be active in chronic human liver disease (Figure 7F). In addition, human phase I and phase II metabolizing enzymes showed complex expression, changes with the strongest upregulation for the flavin-dependent monooxygenase *FMO1* and the aldehyde dehydrogenase *ALDH3A1* (Supplementary Figure S15). Thus, in both species, complex up- and downregulations of drug metabolizing enzymes were observed, and in several enzyme families, a mouse–human gene-by-gene comparison showed statistically significant correlations.

## 4 Discussion

In the present study, we analyzed metabolic and pharmacokinetic changes in mice with CCl_4_-induced liver cirrhosis through iterative cycles of experimental studies and computational simulations. A major alteration found in the present work is the substantial increase in glucuronides in the blood of cirrhotic mice, which is explained by high levels of the sinusoidal export carriers *Abcc3* and *Abcc4* that are known to be upregulated in cholestatic liver disease and act by exporting bile acids from hepatocytes to avoid an increase beyond cytotoxic levels (Vartak et al., 2016; Jansen et al., 2017; Ghallab et al., 2019b). Increased blood concentrations of these glucuronides lead to increased urinary excretion. The mouse model used in this study with CCl_4_ administered for 1 year underwent severe cholestasis with increased *Abcc3* and *Abcc4* levels, which corresponds to the findings of previous studies (Jansen et al., 2017; Ghallab et al., 2019b). Increased expression of Abcc3 and Abcc4 is, thus, in agreement with the observation in earlier studies in primary biliary cirrhosis (Chai et al., 2012) and acetaminophen-induced liver failure (Barnes et al., 2007; Campion et al., 2008). A consequence of the upregulation of these sinusoidal export carriers is not only the increased export of bile acids from hepatocytes into the sinusoidal blood but also of specific drug glucuronides. According to the PBPK model, increased UGT activity and decreased biliary secretion would, in principle, also explain the increase in blood glucuronides; however, these possible mechanisms were excluded by experimental data since glucuronidation activity was not increased in the microsomes of cirrhotic mouse livers, and the biliary export of glucuronides was only slightly decreased in cirrhotic compared to control mice, and not significant.

A surprising result was the robustness of phase I metabolism in cirrhotic mice, exemplified by the metabolism of caffeine to paraxanthine by the phase I enzyme *Cyp1a2* in healthy mice. Although the expression of *Cyp1a2* was significantly downregulated and the pericentral lobular area with hepatocytes expressing *Cyp1a2* was decreased by almost 90% in cirrhotic mouse livers, the formation of paraxanthine was not decreased in cirrhosis, and the AUC of the parent compound caffeine even decreased. This result was unexpected since we previously performed a study with only a single dose of CCl_4_ that caused acute liver damage (Schenk et al., 2017). During this phase of acute liver damage, the functional impact, as evidenced by the reduced formation of paraxanthine and increased AUC of caffeine, was even more than the model prediction based on the loss of CYP1A2-expressing hepatocytes (Schenk et al., 2017). Thus, a single dose of CCl_4_ greatly compromised the phase I metabolism of caffeine. In contrast, long-term administration of CCl_4_, as performed in the present study, led to a situation where phase I metabolism of caffeine was not reduced despite a massive decrease in *Cyp1a2* expression. This conundrum may be explained by the upregulation of other enzymes with similar metabolic activity in cirrhotic livers that can compensate for the downregulated CYPs. Genome-wide analysis of mouse livers after 1 year of CCl_4_ showed that besides downregulation, several CYPs were also induced. Moreover, other phase I enzymes, such as flavin monooxygenases (FMO), were also upregulated in cirrhotic mouse livers. However, since so many phase I enzymes are induced in the cirrhotic liver, the question of which isoenzymes are relevant for the substrates investigated in this study was not addressed in the present study. Nevertheless, the observation that the phase I metabolizing capacity of microsomal preparations of cirrhotic livers was not reduced compared to that of vehicle controls suggests that the transcriptionally upregulated phase I enzymes indeed functionally compensate for the loss of predominantly pericentral CYPs (Supplementary Figure S16). This putative backup function is in line with previous analyses, suggesting a constitutive role of FMOs in drug metabolism (Phillips and Shephard, 2017).

At first glance, the expression changes of CYPs discussed in the previous paragraph may appear surprising since the mRNA expression of some CYPs, such as *Cyp1a2* and *Cyp2c29*, decreases. In contrast, the expression of other Cyp isoforms, such as *Cyp3a11* and *Cyp2d22*, remained unchanged. A possible explanation for this difference may be the zonation of some CYPs; for example, *Cyp2e1*, an enzyme that metabolically toxifies CCl_4_, is expressed in the pericentral lobular zone. Therefore, it appears plausible that other enzymes with a pericentral expression pattern decrease upon CCl_4_ exposure, while enzymes that are expressed uniformly over the lobule are less affected or even become upregulated to compensate (Supplementary Figure S16).

An important question is if the mechanisms observed in mice in the present study are also relevant in humans with chronic liver disease. In agreement with previous studies, the present data show that human hepatocytes adapt to cholestasis via similar mechanisms as in mice, namely, by reduced uptake of bile acids from sinusoidal blood and increased export from hepatocytes into the sinusoids (Dawson et al., 2009; Jansen et al., 2017). The database investigated in this study with expression data from liver tissue of patients with chronic liver disease showed, for example, that *SLC51B*, a carrier that exports taurine conjugates of bile acids from hepatocytes into sinusoidal blood, is upregulated in chronic liver disease. In addition, *SLC10A1* (*NTCP*), a major sinusoidal bile acid uptake carrier, was downregulated in the datasets of Hoang and Ramnath (Holland et al., 2022), which is in agreement with previous reports (Dawson et al., 2009). Therefore, hepatocytes from both species seem to protect themselves against bile acid overload using comparable mechanisms, which may lead to similar consequences for drug metabolism, such as enhanced sinusoidal export of some drug glucuronides.

In conclusion, the present study shows that liver cirrhosis leads to increased levels of glucuronides of several drugs in the blood due to the upregulation of sinusoidal export carriers, which leads to increased urinary excretion. Moreover, phase I metabolism in chronic liver disease is surprisingly well-compensated, which is in contrast to the situation in acute injury.

## Data Availability

The original contributions presented in the study are included in the article/Supplementary Material; further inquiries can be directed to the corresponding authors.
