# Supplementary material for: Increased sinusoidal export of drug glucuronides is a compensative mechanism in liver cirrhosis of mice
Source: Front Pharmacol. 2023 Nov 20;14:1279357. doi: 10.3389/fphar.2023.1279357 (PMC10694292; doi:10.3389/fphar.2023.1279357)
Supplement: Supplementary file 1 [file DataSheet1.PDF]

Supplementary Material

1. Immunohistochemistry

Table S1. Concentrations of primary and secondary antibodies

Antigen	Tissue	Primary antibodies		Secondary antibodies	
		Antibody	Concentration	Antibody	Concentration
CD45	FFPE	Rat anti-CD45	1:400	Rabbit anti-rat IgG	1:1000
CK19	FFPE	Rabbit anti-CK19	1:500	Swine anti-rabbit	1:20
CYP3A	Frozen	Rabbit anti-Cyp3a1	1:250	Swine anti-rabbit	1:20
CYP1A	Frozen	Rat anti-Cyp1a2	1:500	Rabbit anti-rat IgG	1:1000
CYP2C	Frozen	Rat anti-Cyp2c6	1:250	Rabbit anti-rat IgG	1:1000
CYP2E1	FFPE	Rabbit anti-Cyp2e1	1:100	Swine anti-rabbit	1:20
FSP1	FFPE	Rabbit anti-S100A4	1:4000	Swine anti-rabbit	1:20
GS	FFPE	Mouse anti-GS	1:1000	Anti-mouse	1:500

2. Determination of microsomal CYP and UGT enzyme activation

Table S2: Incubation conditions for determination of microsomal CYP and UGT enzyme activities

Substrate	Concentration (µM)	Incubation time (min)
CYP activity		
caffeine	50	20
codeine	50	20
midazolam	5	10
torsemide	20	60
Glucuronidation activity		
codeine	1400	30
morphine	250	20
1-hydroxymidazolam	40	30

3. PBPK model development

3.1. Caffeine

Active Transport

Active transport is not relevant for caffeine and paraxanthine.

Enzymatic Clearance

Caffeine is primarily eliminated via hepatic metabolism. In humans, it is a probe drug for CYP1A2 activity and paraxanthine is the main metabolite (Nehlig, 2018). The murine homologue of *CYP1A2* is *Cyp1a2* and responsible for 88% of the systemic caffeine clearance in C57BL/6N mice (Buters et al., 1996). Like human CYP1A2, murine CYP1A2 is primarily expressed in the liver (Zanger and Schwab, 2013;Hersman and Bumpus, 2014). However, the main metabolite of caffeine in mice is theobromine, not paraxanthine (Bonati et al., 1985) and the amounts of paraxanthine formed in murine liver microsomes are smaller compared to human liver microsomes (Labedziki et al., 2002). Paraxanthine formation is the only metabolic clearance process for caffeine in the reference model because the optimized rate of an additional total hepatic clearance process was zero. Thus, the total hepatic clearance process for caffeine was removed. Paraxanthine clearance was described as a total hepatic clearance process because no further metabolites were measured.

Renal clearance

Renal excretion was considered for caffeine and paraxanthine. Only a small fraction of caffeine is excreted unchanged to urine, about 0.5-2% were reported in humans(Nehlig, 2018) and about 5% reported in mice (Bonati et al., 1985). The mean fraction of dose found after the administration of the drug cocktail was 1% for caffeine and 2% for paraxanthine.

Biliary Clearance

Biliary clearance of paraxanthine and caffeine not mentioned in the literature and therefore not considered in the PBPK model. Measurement of biliary excretion yielded a mean fraction of dose of 1.93% for caffeine and 0.08% for paraxanthine 2h after drug administration.

Model Building

In addition to the clearance parameters, the fu of caffeine was optimized because kinetics of caffeine could not be well described with the human fu. Drug protein binding is species specific and the fu in mice is often higher than in humans. The optimized mouse fu (0.79) is higher than the human fu (0.64(Blanchard, 1982)) for caffeine and lower than the fu of the metabolite paraxanthine (0.85(Bortolotti et al., 1985)) which indicates a reasonable fit.

Parameters

Table S3: Caffeine parameters

**Caffeine**	**Reference Model Parameter**	**Source**	**Literature Values, Comment**
Lipophilicity	-0.07	DrugBank(Wishart et al., 2017) (experimental)	
Fraction Unbound	0.79	optimized	0.70(Thiel et al., 2015) (mouse, optimized)<br>0.64(Blanchard, 1982) (human, measured)
Molecular Weight	194.2 g/mol	DrugBank(Wishart et al., 2017)	
pKa	10.4 (Base)	DrugBank(Wishart et al., 2017)	
Solubility	21600 mg/l	DrugBank(Wishart et al., 2017)	
Partition Coefficients	PK-Sim Standard		
Cellular Permeabilities	PK-Sim Standard		
Metabolizing Enzyme	Cyp1a2	optimized	PKSim Mouse DB Version 7
Caffeine -> Paraxanthine	Specific clearance<br>1.51*10^-2 1/min		
GFR Fraction	2.52*10^-3	optimized	
Biliary Clearance	NA	NA	
Total Hepatic Clearance	NA	NA	

Table S4: Paraxanthine parameters

**Paraxanthine (1,7-Dimethylxanthine)**	**Reference Model Parameter**	**Source**	**Literature Values, Comment**
Lipophilicity	-0.63	DrugBank(Wishart et al., 2017) (AlogPS prediction)	0.27(Wishart et al., 2017) (ChemAxon prediction)
Fraction Unbound	0.85	Bortolotti 1985(Bortolotti et al., 1985) (rat,measured)	0.56(Lelo et al., 1986) (human, measured)
Molecular Weight	180.2 g/mol	DrugBank(Wishart et al., 2017)	
pKa	Neutral	FooDB	
Solubility	9130 mg/l	DrugBank(Wishart et al., 2017) (AlogPS prediction)	
Partition Coefficients	PK-Sim Standard		
Cellular Permeabilities	PK-Sim Standard		
Metabolizing Enzyme	NA	NA	
GFR Fraction	1.12*10^-2		
Biliary Clearance	NA	NA	
Total Hepatic Clearance	0.10 1/min	optimized	

3.2. Codeine

Active Transport

Active transport of the glucuronidated metabolites M3G and C6G was required to achieve a good model fit. Glucuronides are more hydrophilic than the parent drug and negatively charged at physiological pH, therefore they mostly can not cross membranes passively (Zamek-Gliszczynski et al., 2006;Järvinen et al., 2017). The charge dependent Schmitt method was chosen for the calculation of cellular permeabilities because it considers the pKa, which leads to a much lower cellular permeability compared to the PK-Sim Standard method.

MRP3 transports M3G (Zelcer et al., 2005;van de Wetering et al., 2007) and is the most relevant basolateral efflux transporter for glucuronides (Zamek-Gliszczynski et al., 2006). Therefore, MRP3 also transports C6G in the PBPK model.

Active transport of the other molecules was not considered, although morphine is a substrate of OCT1 (SLC22A1) in humans and transporter genotype can influence pharmacokinetic parameters (Tzvetkov et al., 2013).

Enzymatic Clearance

Cyp3a11 and *Cyp2d22* are the murine homologues of *CYP3A4* and *CYP2D6* respectively (Martignoni, 2006). It was assumed that the murine enzymes catalyze the same reactions in mice as their homologues do in humans although there is a report that suggests different CYP2D22 substrate specificities (Yu and Haining). UGT2B36 metabolizes Morphine to M3G (Kurita et al., 2017), therefore it was assumed that UGT2B36 also catalyzes the formation of C6G.

Renal Clearance

Renal clearance of codeine and all metabolites was described as a glomerular filtration process. Mean fractions of dose excreted to urine were 3% (Codeine), 1% (C6G), 3% (Norcodeine), 1% (Morphine) and 13% (M3G).

Biliary Clearance

In the drug cocktail PK study, experimentally observed fractions excreted to bile were below 1% for codeine and all metabolites. Nevertheless, biliary clearance of M3G and C6G were considered in some model structures for the hypothesis testing. Previous studies reported higher metabolite amounts in bile. Isolated perfused rat liver experiments showed that a substantial fraction of M3G (~0.3-0.5) formed in the hepatocytes was excreted to bile(Evans et al., 1999). Another study showed that two hours after morphine administration to mice, about 30-50% of the dose were excreted to bile as M3G(van de Wetering et al., 2007).

Model Building

In addition to the clearance parameters, lipophilicity of codeine was optimized because codeine plasma concentrations could not be described using the experimentally determined lipophilicity.

The codeine PBPK model comprises the parent drug and four metabolites, three drug metabolizing enzymes, one drug transporter as well as renal and biliary clearances. The comparably large number of unknown process parameters made parameter fitting in PK-Sim challenging, therefore the number of optimized molecule parameters was reduced to the absolute minimum. Lipophilicities for metabolites were not optimized, it was possible to achieve a good model fit with published values even though some of them were only in silico predictions.

Fractions unbound were also not optimized although they reflect human parameter values. Codeine and its metabolites have relatively high unbound fractions in humans and murine unbound fractions are typically higher. For C6G, a literature value was missing, an unbound fraction of 1 was assumed because the measured fraction unbound of the other glucuronidated metabolite M3G was 0.9. For parameter fitting, the weights of the urine measurements were increased to 2.5 to achieve a good fit.

Parameters

Table S5: Codeine parameters

**Codeine**	**Reference Model Parameter**	**Source**	**Literature Values, Comment**
Lipophilicity	2.16	optimized	1.39(Wishart et al., 2017) (experimental) 1.19(Avdeef, 1996) (experimental)
Fraction Unbound	0.75	DrugBank(Wishart et al., 2017) (human)	0.44(Vree and Verwey-van Wissen) (experimental, human)
Molecular Weight	299.4 g/mol	DrugBank(Wishart et al., 2017)	
pKa	Neutral	Assumption	Base: 8.22(Avdeef, 1996)
Solubility	1mg/ml	Assumption	not rate limiting
Partition Coefficients	Schmitt(Schmitt, 2008)		
Cellular Permeabilities	Charge dependent Schmitt		
Metabolizing Enzyme	Cyp2d22		Expression from PKSim Mouse DB Version 7
Codeine -> Morphine	Specific clearance: 6.56*10^-2 1/min	optimized	
Metabolizing Enzyme	Cyp3a11		Expression from PKSim Mouse DB Version 7
Codeine -> Norcodeine	Specific clearance: 12.64*10^-2 1/min	optimized	
Metabolizing Enzyme	Ugt2b36		Expression from PKSim Mouse DB Version 7
Codeine -> C6G	Specific clearance: 12.45*10^-2 1/min	optimized	
GFR Fraction	7.6*10^-2	optimized	
Biliary Clearance	NA	NA	
Total Hepatic Clearance	NA	NA	

Table S6: Codeine-6-glucuronide parameters

**Codeine-6-Glucuronide (C6G)**	**Reference Model Parameter**	**Source**	**Literature Values, Comment**
Lipophilicity	-0.23	DrugBank(Wishart et al., 2017) (predicted, AlogPs)	-2.8(Wishart et al., 2017) (predicted, ChemAxon)
Fraction Unbound	1	Assumption	0.44(Vree and Verwey-van Wissen) (experimental, human)
Molecular Weight	475.5 g/mol	DrugBank(Wishart et al., 2017)	
pKa	2.86	Assumption based on M3G pKa of 2.86	
Solubility	4.31 mg/ml	DrugBank(Wishart et al., 2017) (predicted, AlogP)	
Partition Coefficients	Schmitt(Schmitt, 2008)		
Cellular Permeabilities	Charge dependent Schmitt		
Metabolizing Enzyme	NA	NA	
GFR Fraction	0.21	optimized	
Biliary Clearance	160.8 1/min	optimized	
Total Hepatic Clearance	NA	NA	
Transport Proteins<br>ABCC3	Km: 1µmol/l<br>Vmax: 5.19 µmol/l/min	Assumption<br>optimized	Expression from PKSim Mouse DB Version 7

Table S7: Norcodeine parameters

Norcodeine	Reference Model Parameter	Source	Literature Values, Comment
Lipophilicity	0.99	DrugBank (Wishart et al., 2017) (predicted, AlogPs)	0.96(Wishart et al., 2017) (predicted, ChemAxon)<br><br>0.69(Avdeef, 1996) (experimental)
Fraction unbound	0.765	Vree 1992(Vree and Verwey-van Wissen) (human)	
Molecular Weight	285.3 g/mol	DrugBank(Wishart et al., 2017)	
pKa	Neutral	Assumption	Base: 9.22(Avdeef, 1996) (experimental)
Solubility	1 mg/ml	Assumption	
Partition Coefficients	Schmitt		
Cellular Permeabilities	Charge dependent Schmitt		
Metabolizing Enzyme	NA		
GFR Fraction	0.30	optimized	
Biliary Clearance	NA		
Total Hepatic Clearance	0.61 1/min	optimized*	

* Accounts for glucuronidation of norcodeine to norcodeine-6-glucuronide

Table S8: Morphine parameters

**Morphine**	**Reference Model Parameter**	**Source**	**Literature Values, Comment**
Lipophilicity	0.87	DrugBank (Wishart et al., 2017) (experimental)	0.89(Avdeef, 1996) (logP uncharged)
Fraction Unbound	0.75 (Albumin)	Edginton 2006(Edginton et al., 2006) (human)	0.535(Vree and Verwey-van Wissen)<br><br>(experimental, human)
Molecular Weight	285.34 g/mol (no Halogens)		
pKa	Neutral	Assumption	Basic pKa: 8.18(Avdeef, 1996)<br><br>Acidic pKa: 9.26 (Avdeef, 1996)<br><br>8.21(Poulin and Theil, 2002)
Solubility	249 mg/l	DrugBank(Wishart et al., 2017) (experimental)	
Partition Coefficients	Schmitt(Schmitt, 2008)		
Cellular Permeabilities	Charge dependent Schmitt		
Metabolizing Enzymes	Ugt2b36		Expression from PKSim Mouse DB Version 7
Morphine-> M3G	Specific clearance:<br><br>30.39 1/min	optimized	
GFR Fraction	1	optimized*	

Biliary Clearance	NA	NA
Total Hepatic Clearance	NA	NA

* The physiological upper boundary of the GFR fraction is one. Higher fractions indicate active secretion of morphine by drug transporters, which were not considered in the model.

Table S9: Morphine-3-glucuronide parameters

Morphine-3-Glucuronide (M3G)	Reference Model Parameter	Source	Literature Values, Comment
Lipophilicity	-1.1	Avdeef 1996(Avdeef, 1996)	
Fraction Unbound	0.9	DrugBank(Wishart et al., 2017)	
Molecular Weight	461.47 g/mol	PubChem(National Center for Biotechnology Information, 2022b)	
pKa	Acid: 2.86	Avdeef 1996(Avdeef, 1996)	
Solubility	1.15 mg/ml	DrugBank(Wishart et al., 2017)	
Partition Coefficients	Schmitt		
Cellular Permeabilities	Charge dependent Schmitt		
Metabolizing Enzyme	NA	NA	
GFR Fraction	0.12	optimized	
Biliary Clearance	0	optimized	
Total Hepatic Clearance	NA	NA	
Transport Proteins<br>ABCC3	Km: 1µmol/l<br>Vmax: 999.44 µmol/l/min	assumption<br>optimized	Expression from PKSim Mouse DB Version 7

Table S10: Overview of metabolic reactions in the codeine PBPK model

**Substrate**	**Metabolite**	**Enzyme/Process**	**Human homologue**	**Reference**
Codeine	C6G	Ugt2b36	UGT2B7	Assumption based on Kurita 2017
Codeine	Norcodeine	Cyp3a11	CYP3A4	
Codeine	Morphine	Cyp2d22	CYP2D6	
Morphine	M3G	Ugt2b36	UGT2B7	Kurita 2017
Norcodeine	Not modeled	Total hepatic Clearance		

3.3. Midazolam

Active Transport

Active transport is not relevant for midazolam (MDZ) and 1-hydroxymidazolam (OH-MDZ), but for OH-MDZ-G. Glucuronides are more hydrophilic than the parent drug and negatively charged at physiological pH, therefore they mostly can not cross membranes passively (Zamek-Gliszczynski et al., 2006;Järvinen et al., 2017). The Schmitt(Schmitt, 2008) method was chosen for the calculation of cellular permeabilities because it considers the pKa, which leads to a much lower cellular permeability compared to the PK-Sim Standard method.

The glucuronide transporter MRP3 was considered in the midazolam PBPK model because it is considered the most relevant basolateral efflux transporter for glucuronides(Zamek-Gliszczynski et al., 2006).

Enzymatic Clearance

Midazolam is primarily eliminated via metabolism and a probe drug for CYP3A4 in humans. *Cyp3a11* is the mouse gene that is most similar to human *CYP3A4* expressed predominantly in the liver, but also in the intestine(Martignoni et al., 2006;Hart et al., 2008;Renaud et al., 2011).

Formation of 1’OH midazolam as a product of midazolam hydroxylation by CYP3A11 was considered in the model. However, CYP2C enzymes might metabolize midazolam in mice as well (Perloff et al., 200;van Waterschoot et al., 2007).

A total hepatic clearance process of midazolam was included in the model to account for the formation of other metabolites such as 4’OH-Midazolam.

UGT2B36 catalyzes the glucuronidation of 1’OH-Midazolam in the PBPK model. In human liver microsomes, UGT2B4/2B7 are the relevant isoforms(Seo et al., 2010). Murine *Ugt2b36* is homologue to human *UGT2B7*, the protein sequence identity is 67%.

Renal Clearance

Renal clearance was only considered for 1’OH-MDZ-G. The fractions of dose excreted to urine of MDZ and 1’OH-MDZ after administration of the drug cocktail were far below 1%.

Biliary Clearance

Biliary clearance of OH-MDZ-G was considered in the model. The mean observed fraction excreted to bile was 1.2 % 2 h after drug administration.

Model Building

Lipophilicity, fu and pKa of OH-MDZ-G were unknown. Lipophilicity was assumed to be 1.4 which represents the mean difference of the lipophilicities of other hydroxylated compounds and their corresponding glucuronides (Vossen et al., 2007).

The human fu (0.03) of OH-MDZ-G assumed by Vossen et al.(Vossen et al., 2007) appears too low for mice. The fu of the phase I metabolite 1’OH-MDZ is already much higher (fu = 0.15). Glucuronides

are usually more hydrophilic and therefore exhibit lower protein binding than phase I metabolites. Therefore, parameter identification boundaries for the fu of OH-MDZ-G were 0.15 - 1.

The pKa of OH-MDZ-G is assumed to be 2.86 because the pKa of another the phase 2 metabolite M3G is 2.86. The carboxy group of UDP glucuronic acid is preserved upon conjugation, thus OH-MDZ-G is acidic.

For parameter fitting, the weights of the urine measurements were increased to 5 to achieve a good fit to the urine fraction.

Parameters

Table S11: Midazolam parameters

Midazolam	Reference Model Parameter	Source	Literature Values, Comment
Lipophilicity	2.95	Poulin 2002(Poulin and Theil, 2002) (measurement)	3.89 (Wishart et al., 2017) (prediction)<br>3.13(Britz et al., 2019) (optimized)<br>3.33(Schenk et al., 2017) (optimized)
Fraction unbound	0.08	Varkhede 2018(Varkhede et al., 2018) (measurement, mouse)	0.03(Wishart et al., 2017) (human)<br>0.046 (Maurer et al., 2005;Schenk et al., 2017)<br>0.027(Poulin and Theil, 2002) (rat)
Molecular Weight	325.77 g/mol<br>(Has Halogens: 1F, 1Cl)		
pKa	Base: 6.57	DrugBank(Wishart et al., 2017)	5.82(Poulin and Theil, 2002) (experimental)<br>6.04(Nguyen et al., 2016) (prediction)<br>6.14 (Schenk et al., 2017)
Solubility	0.05 mg/ml (Ref pH: 6.5)	Heikkinen 2012(Heikkinen et al., 2012)	
Partition Coefficients	Schmitt(Schmitt, 2008)		

Cellular Permeabilities	Charge dependent Schmitt		
Metabolizing Enzymes	Cyp3a11		Expression from PKSim Mouse DB Version 7
MDZ-> 1'OH-MDZ	Specific clearance:<br>2.83 1/min	optimized	
GFR Fraction	NA	NA	
Biliary Clearance	NA	NA	
Total Hepatic Clearance	9.48 1/min	optimized	

Table S12: 1-Hydroxymidazolam parameters

**1-Hydroxymidazolam (OH-MDZ)**	**Reference Model Parameter**	**Source**	**Literature Values, Comment**
Lipophilicity	2.48	DrugBank(Wishart et al., 2017) (predicted, ChemAxon)	3.09(Wishart et al., 2017) (predicted AlogPS)<br>2.5(Nguyen et al., 2016) (predicted, ACD)<br>2.6 (Vossen et al., 2007)
Fraction Unbound	0.15	Nguyen 2016(Nguyen et al., 2016) (measured, human)	0.01(Schenk et al., 2017) (optimized, mouse)
Molecular Weight	341.77g/mol<br>(Has Halogens: 1F, 1Cl)	DrugBank(Wishart et al., 2017)	
pKa	Neutral	Assumption	Ampholyte<br>3.63, 13.6(Nguyen et al., 2016) (predicted ACD)<br>4.99, 13.95 (Wishart et al., 2017) (predicted)
Solubility	0.0254 mg/ml	DrugBank(Wishart et al., 2017) (prediction, AlogPS)	
Partition Coefficients	Schmitt(Schmitt, 2008)		
Cellular Permeabilities	Charge dependent Schmitt		

Metabolizing Enzymes	Ugt2b36		Expression from PKSim Mouse DB Version 7
OH-MDZ -> OH-MDZ-G	Specific clearance:<br><br>0.74 1/min	optimized	
GFR Fraction	NA	NA	
Biliary Clearance	NA	NA	
Total Hepatic Clearance	NA	NA	

Table S13: 1-Hydroxymidazolam glucuronide parameters

**1-Hydroxymidazolam glucuronide (OH-MDZ-G)**	**Reference Model Parameter**	**Source**	**Literature Values, Comment**
Lipophilicity	1.4	Vossen 2007(Vossen et al., 2007)	
Fraction Unbound	0.60	optimized	
Molecular Weight	517.9 g/mol<br>(Has Halogens: 1F, 1Cl)	Vossen 2007(Vossen et al., 2007)	
pKa	Acid: 2.86	Assumption based on M3G pKa of 2.86	
Solubility	1 mg/ml	Assumption	PK-Sim default
Partition Coefficients	Schmitt(Schmitt, 2008)		
Cellular Permeabilities	Charge dependent Schmitt		
Metabolizing Enzyme	NA	NA	
GFR Fraction	2.02	optimized	
Biliary Clearance	47.33 1/min	optimized	
Total Hepatic Clearance	NA	NA	
Transport Proteins<br>ABCC3	Km: 1µmol/l<br>Vmax: 9.15 µmol/l/min	Assumption<br>optimized	Expression from PKSim Mouse DB Version 7

3.4. Pravastatin

Active Transport

Active transport affects pravastatin disposition in humans. *Oatp1b2* is the mouse homologue to human *OATP1B1*. Knockout of *Oatp1b2* altered pravastatin pharmacokinetics in mice (Zaher et al., 2008). Therefore, pravastatin transport by OATP1B2 was considered in the PBPK model.

Enzymatic Clearance

CYP-mediated metabolism does not significantly contribute to pravastatin clearance, therefore it was not considered in the PBPK model.

Renal Clearance

Less than 1% of the pravastatin dose was recovered unchanged from urine. Nevertheless, a renal clearance process was considered in the PBPK model. In humans, about 41% of the dose could be recovered unchanged from urine(Everett et al., 1991).

Biliary Clearance

Biliary clearance of pravastatin constitutes the most important elimination pathway. The mean fraction of dose excreted to bile 2h postdose was 41%. In rats, about 60% of the dose were found in bile (Fukuda et al., 2008).

Model Building

In addition to the clearance parameters, lipophilicity was optimized because different parameter values were reported in the literature.

Parameters

Table S14: Pravastatin parameters

Pravastatin	Reference Model Parameter	Source	Literature Values, Comment
Lipophilicity	0.12	optimized	-0.23(Serajuddin et al., 1991) (experimental pH=7)<br>1.6(Serajuddin et al., 1991)(experimental pH=5)<br>0.59(Wishart et al., 2017) (MSDS)<br>0.82(Lippert et al., 2012) (human,optimized)
Fraction Unbound	0.672	Fukuda 2008(Fukuda et al., 2008)(rat)	0.52-0.57(Wishart et al., 2017)(human)<br>0.5(FDA, 2020)<br>0.73(Thiel et al., 2015) (mouse, optimized)
Molecular Weight	424.53 g/mol	DrugBank(Wishart et al., 2017)	
pKa	Acid: 4.2	DrugBank(Wishart et al., 2017), Puttegowda 2016(Puttegowda et al., 2016)	
Solubility	10 mg/ml	DrugBank(Wishart et al., 2017)	
Partition Coefficients	PK-Sim Standard		

Cellular Permeabilities	PK-Sim Standard		
Metabolizing Enzyme	NA	NA	
GFR Fraction	4.66*10^-2	Optimized	
Biliary Clearance	208.13 1/min	optimized	
Total Hepatic Clearance	NA	NA	
Transport Proteins<br><br>Oatp1b2	Km= 1µmo/l<br><br>Vmax: 52.36 µmol/l/min	Assumption<br><br>optimized	Oatp1b2 expression from PKSim Mouse DB Version 7<br><br>Km: 11.5 µmol/L(Nakai et al., 2001)(human, experimental)<br><br>Vmax: 2.5 µmol/L/min(Thiel et al., 2015)<br><br>(optimized for mice with measured human Km)

3.5. Talinolol

Active transport

Talinolol is a probe drug for MDR1 activity in humans(Matthaei et al., 2016). *Mdr1a* and *Mdr1b* are the two murine homologues of human *MDR1*. Murine MDR1A/B also transports talinolol, functional data from *Mdr1a/1b* knockout mice showed increased talinolol plasma concentrations compared to wildtype mice(Schwarz et al., 2001). In the PBPK model, only MDR1A was considered because MDR1A seems to be more important for drug transport than MDR1B(Collett et al., 2004).

Enzymatic Clearance

In humans, CYP-mediated metabolism does not significantly contribute to talinolol clearance(Oertel et al., 1994), therefore metabolic clearance was not considered in the murine talinolol PBPK model.

Renal Clearance

In humans, renal clearance constitutes the most important elimination process, about 60% of the dose were recovered unchanged from urine(Trausch et al., 1995). Renal clearance seems to be less important for talinolol excretion in mice than in humans. The mean fraction of dose recovered from urine after drug cocktail administration to mice was 4%.

Biliary Clearance

Biliary clearance of talinolol was included in the model, because an additional route of excretion is needed when the drug is not metabolized and urinary excretion is low. However, the mean fraction of dose found in bile 2h after drug cocktail administration was only 0.5%. In humans, the median fraction of dose found in bile after IV administration was 9.3%(Terhaag et al., 1989).

Model Building

In addition to the clearance parameters, talinolol lipophilicity was fitted because reported literature values ranged from 1.08(Gramatté et al., 1996) to 2.9 (DrugBank).

Talinolol has a high pKa and the vast majority of talinolol is charged at physiological pH which reduces the membrane permeability. Cellular permeability calculation methods PK-Sim standard and Charge-dependent Schmitt were tested, the latter considers the pKa. The Schmitt calculation method resulted in 100-fold lower cellular permeabilities. In the talinolol reference model, cellular permeabilities were calculated with the PK-Sim standard method because the fitted lipophilicity was closer to the reported literature values.

Given negligible talinolol metabolism, low renal excretion (<5%), and low biliary excretion (< 1%), there must be an additional elimination mechanism. One possibility is intestinal secretion because talinolol transport against a concentration gradient has been(Trausch et al., 1995;Gramatté et al., 1996;Mutschler et al., 2001). Despite *Mdr1a* (*Abcb1a*) being expressed in the intestinal mucosa compartment of the PBPK model (normalized expression up to 1), intestinal secretion of talinolol to faeces does not take place in the PBPK model. In the PBPK model, biliary and renal excretion account for the complete dose and therefore biliary excretion is strongly overpredicted if no total hepatic clearance process is considered.

Parameters

Table S15: Talinolol parameters

**Talinolol**	**Reference Model Parameter**	**Source**	**Literature Values, Comment**
Lipophilicity	2.74	optimized	1.08(Gramatté et al., 1996) (octanol-water partition coefficient = 12 at pH=7.5)<br><br>2.8(Wishart et al., 2017)(predicted, ChemAxon)<br><br>2.91(Wishart et al., 2017)(predicted, AlogPS)<br><br>2.3(Schenk et al., 2017) (optimized)
Fraction Unbound	0.39	Obach 2008(Obach et al., 2008) (measured, human)	0.45(Tubic et al., 2006) (human, possibly optimized)<br><br>0.65(Thiel et al., 2015) (mouse, possibly optimized)
Molecular Weight	363.5 g/mol	PubChem(National Center for Biotechnology Information, 2022a)	
pKa	Base: 9.3	Matthei 2016(Matthaei et al., 2016)	
Solubility	3.4 mMol/L	Gramatté 1996(Gramatté et al., 1996)	
Partition Coefficients	PK-Sim Standard		
Cellular Permeabilities	PK-Sim Standard		

Metabolizing Enzyme	NA	NA	
GFR Fraction	0	optimized*	
Biliary Clearance	2.30 1/min	optimized	
Total Hepatic Clearance	0	optimized	
Transport Proteins	Km= 1µmo/l	assumption	Expression from PKSim Mouse DB Version 7
Abcb1a/Mdr1a	Vmax= 11.18 µmol/l/min	optimized	

*Transporter expression in the kidney is sufficient to explain the drug fraction in urine

3.6. Torsemide

Permeability & Distribution

Reported tissue-to-plasma ratios for torsemide are low (e.g. liver 0.336, muscle 0.069), which is unusual for a lipophilic drug (Lee 2005). It was not possible to fit the PBPK model with a logPs from the DrugBank database (1.8 or 3.37) and fu (0.06, rat) for torsemide because predicted plasma concentrations were far below observed plasma concentrations. Adjustment of the endothelial permeability was required, endothelial permeability in the liver was set to 10 cm/min and to 0.1 cm/min in all other tissues except for the brain.

Active Transport

Torsemide is substrate of the OATP1B1 transporter in humans. *Oatp1b2* is the mouse homologe of human *OATP1B1(Ogura et al., 2000;Zaher et al., 2008)*. Active transport of torsemide and OH-torsemide by OATP1B2 was tested, but not included in the final model, because the transporter activity (Vmax) did not affect the model simulation and was fitted to 0 or to values close to 0.

Enzymatic Clearance

In humans, hepatic metabolism makes up about 80% of the drug clearance and about 20% are excreted to urine(FDA, 2017).

A homologue to human CYP2C9 missing in mice(Hart et al., 2008) , the expression profile of *Cyp2c29* was chosen instead. CYP2C29 it is predominantly expressed in the liver like the human CYP2C9 and CYP2C19 (Martignoni et al., 2006;Hersman and Bumpus, 2014). *Cyp2c29* is homologue to human *CYP2C19(Hart et al., 2008).*

OH-torsemide concentration after drug cocktail administration was very low compared to the concentration of other metabolites.

Total hepatic clearance processes were considered for torsemide and OH-torsemide to account for metabolites other that were not measured.

Renal Clearance

Renal clearance was considered in the model. Mean fractions of dose excreted to urine were 4% (torsemide) and 11% (OH-torsemide). In humans, about 20% of torsemide and about 10% of OH-torsemide were recovered from urine after a single oral dose (Knauf and Mutschler, 1998;FDA, 2017).

Biliary Clearance

Biliary clearance was not considered, because the relevance of biliary excretion for drug elimination is negligible. The mean observed fractions excreted to bile were 2 % for torsemide and 0.8% for OH-torsemide 2 h after drug administration. In rat, about 2% of the dose were found in bile after 8h cannulation (Lee et al., 2005).

Model Building

It was not possible to fit the PBPK model with a logPs from the DrugBank database (3.37 or 1.8) and fu (0.06, rat) for Torsemide because predicted plasma concentrations were far below observed plasma concentrations. The underestimation of torsemide plasma concentrations indicated a slower tissue uptake in vivo than predicted by the model. The model simulation did not to capture the kinetics even when both parameters were fitted. To reduce the tissue uptake in the PBPK model, the endothelial permeability was reduced as described previously(Thiel et al., 2015;Krauss et al., 2017). The reduction of the endothelial permeability resulted in a good model fit, but also indicated that the processes governing torsemide PK are not fully understood. In humans, torsemide is transported into cells by OATP1B1 but including an uptake transporter into the PBPK model is not reasonable when tissue uptake is rate-limited by the endothelial permeability. Therefore, the OATP1B2 transporter was not represented in the torsemide PBPK model.

Table S16: Torsemide parameters

Torsemide	Reference Parameter	Model	Source	Literature Values, Comment
Lipophilicity	2.47		optimized	3.356(Wishart et al., 2017) (experimental)<br>1.76 /1.86(Wishart et al., 2017) (predicted AlogPS/ChemAxon)<br>2.023(Thiel et al., 2015) (mouse, optimized)<br>2.3(Schenk et al., 2017) (mouse, optimized)<br>0.57(Knauf and Mutschler, 1998)
Fraction unbound	1*10^-3		optimized	0.061(Lee et al., 2005) (experimental, rat)<br><0.01(Knauf and Mutschler, 1998;Wishart et al., 2017) (experimental, human)<br>0.00125(Krauss et al., 2017) (optimized, human)
Molecular Weight	348.42 g/mol		DrugBank(Wishart et al., 2017)	
pKa	Neutral		Assumption	7.1(FDA, 2017;Wishart et al., 2017)<br>7(Knauf and Mutschler, 1998)

Solubility	0.05596 mg/ml	DrugBank(Wishart et al., 2017) (predicted, AlogPS)	
Partition Coefficients	PK-Sim Standard		
Cellular Permeabilities	PK-Sim Standard		
Metabolizing Enzyme	Cyp2c29	optimized	Expression from PKSim Mouse DB Version 7
Torsemide->OH-Torsemide	Specific clearance:<br><br>2.61 1 /min		
GFR Fraction	0.52	optimized	
Biliary Clearance	NA	NA	
Total Hepatic Clearance	0	optimized	
Transport Proteins<br><br>Slco1b2	Vmax = 0	Not included in reference model	Slco1b2 expression from PKSim Mouse DB Version 7

Table S17: OH-Torsemide parameters

OH-Torsemide	Reference Model Parameter	Source	Literature Values, Comment
Lipophilicity	2	optimized	0.86(Wishart et al., 2017) (predicted AlogPS)<br>0.49(Wishart et al., 2017) (predicted ChemAxon)<br>2.139(Krauss et al., 2017) (optimized, human)<br>0.75(Schenk et al., 2017) (optimized, mouse)
Fraction unbound	0.06	optimized	0.00191(Krauss et al., 2017) (optimized, human)<br>0.01(Schenk et al., 2017) (assumption, mouse)
Molecular Weight	364.41 g/mol	DrugBank(Wishart et al., 2017)	
pKa	Neutral	assumption	
Solubility	0.0898 mg/ml	DrugBank(Wishart et al., 2017) (predicted, AlogPS)	
Partition Coefficients	PK-Sim Standard		
Cellular Permeabilities	PK-Sim Standard		
Metabolizing Enzyme	NA	NA	

GFR Fraction	2.70	optimized	
Biliary Clearance	NA	NA	
Total Hepatic Clearance	3.25 1/min	optimized	
Transport Proteins<br><br>Slco1b2	Km= NA<br><br>Vmax = NA	Not included in reference model	Expression from PKSim Mouse DB Version 7

3.7. Enzyme expression

Table S18: Enzyme expression

**Tissue**	***Cyp1a2*** **relative expression**	***Cyp2d22*** **relative expression**	***Cyp2c29*** **relative expression**	***Cyp3a11*** **relative expression**	***Ugt2b36*** **relative expression**
**Organs, tissues & matrices**					
Bone	6.26*10^-3	0.02	2.61*10^-3	3.74*10^-3	1.60*10^-3
Brain	7.74*10^-3	0.13	1.77*10^-3	7.29*10^-3	1.22*10^-3
Fat	6.62*10^-3	0.13	1.09*10^-3	4.48*10^-3	1.29*10^-3
Gonads	5.01*10^-3	0.12	1.67*10^-3	4.17*10^-3	8.40*10^-4
Heart	0.01	0.13	2.23*10^-3	8.44*10^-3	2.92*10^-3
Kidney	7.37*10^-3	0.07	3.47*10^-3	0.01	2.88*10^-3
Liver Periportal	1	1	1	1	1
Liver Pericentral	1	1	1	1	1
Lung	0.02	0.24	0.03*10^-3	0.06	0.02
Muscle	9.69*10^-3	0.09	2.35*10^-3	8.04*10^-3	2.4*10^-3
Pancreas	7.89*10^-3	0.06	1.25*10^-3	4.73*10^-3	1.85*10^-3
Skin	8.74*10^-3	0.05	4.35*10^-3	5.32*10^-3	1.73*10^-3
Spleen	6.17*10^-3	0.06	4.57*10^-4	4.65*10^-3	9.65*10^-5
**Intestinal mucosa**					
Duodenum	7.69*10^-3	0.07	8.78*10^-3	0.14	9.68*10^-3
Upper Jejunum	7.69*10^-3	0.07	8.78*10^-3	0.14	9.68*10^-3

Lower Jejunum	7.69*10^-3	0.07	8.78*10^-3	0.14	9.68*10^-3
Upper Ileum	7.69*10^-3	0.07	8.78*10^-3	0.14	9.68*10^-3
Lower Ileum	7.69*10^-3	0.07	8.78*10^-3	0.14	9.68*10^-3
Cecum	8.21*10^-3	0.07	7.27*10^-4	4.44*10^-3	4.4*10^-3
**GIT – Non-mucosal tissue**					
Stomach	0.01	0.06	1.21*10^-3	4.36*10^-3	4.51*10^-3
Small Intestine	7.71*10^-3	0.07	0.02	0.27	0.01
Large Intestine	8.21*10^-3	0.07	7.27*10^-4	4.44*10-^3	4.4*10^-3

Tissues without expression were not listed (e.g. the vascular system)

3.8. Transporter expression

Table S19: Transporter expression

Tissue	*Slco1b2* (Influx)	*Abcb1a* (Efflux)	*Abcc3* (Efllux)
**Organs, tissues & matrices**			
Bone	2.44*10^-3	7.74*10^-3	0.13
Brain	1.59*10^-3	0.05	0.03
	(Blood Brain Barrier)	(Blood Brain Barrier)	(Blood Brain Barrier)
Fat	6.11*10^-4	0.05	0.08
Gonads	1.47*10^-3	0.04	0.28
Heart	3.71*10^-3	0.05	0.02
Kidney	2.32*10^-3	0.03 (Apical)	0.06 (Basolateral)
Liver Periportal	1	9.77*10^-3 (Apical)	1 (Basolateral)
Liver Pericentral	1	9.77*10^-3 (Apical)	1 (Basolateral)
Lung	0.02	0.08	0.2
Muscle	1.92*10^-3	0.06	0.05
Pancreas	1.13*10^-3	0.01	0.06
Skin	1.78*10^-3	0.02	0.07
Spleen	3.18*10^-3	0.04	0.15
**Intestinal mucosa**			
Duodenum	2.23*10^-3 (Apical)	0.66 (Apical)	0.5 (Basolateral
Upper Jejunum	2.23*10^-3 (Apical)	0.66 (Apical)	0.5 (Basolateral)

Lower Jejunum	2.23*10^-3 (Apical)	0.66 (Apical)	0.5 (Basolateral)
Upper Ileum	2.23*10^-3 (Apical)	0.66 (Apical)	0.5 (Basolateral)
Lower Ileum	2.23*10^-3 (Apical)	0.66 (Apical)	0.5 (Basolateral)
Cecum	2.23*10^-3 (Apical)	1 (Apical)	0.65 (Basolateral)
**GIT – Non-mucosal tissue**			
Stomach	2.01*10^-3	0.48	0.66
Small Intestine	2.14*10^-3	0.33	0.35
Large Intestine	2.32*10^-3	1	0.65

Tissues without expression were not listed.

Table S20: Reference concentrations and half-lifes

	Cyp1a2	Cyp2d22	Cyp2c29	Cyp3a11	Ugt2b36	Slco1b2	Abcb1a	Abcc3
**Reference Concentration (µmol/l)**	1.80	1	1	1	1	1	1	1
**t1/2 (liver)**	39	36	36	36	36	36	36	36
**t1/2(intestine)**	23	23	23	23	23	23	23	23

4. Ref2erence PBPK models

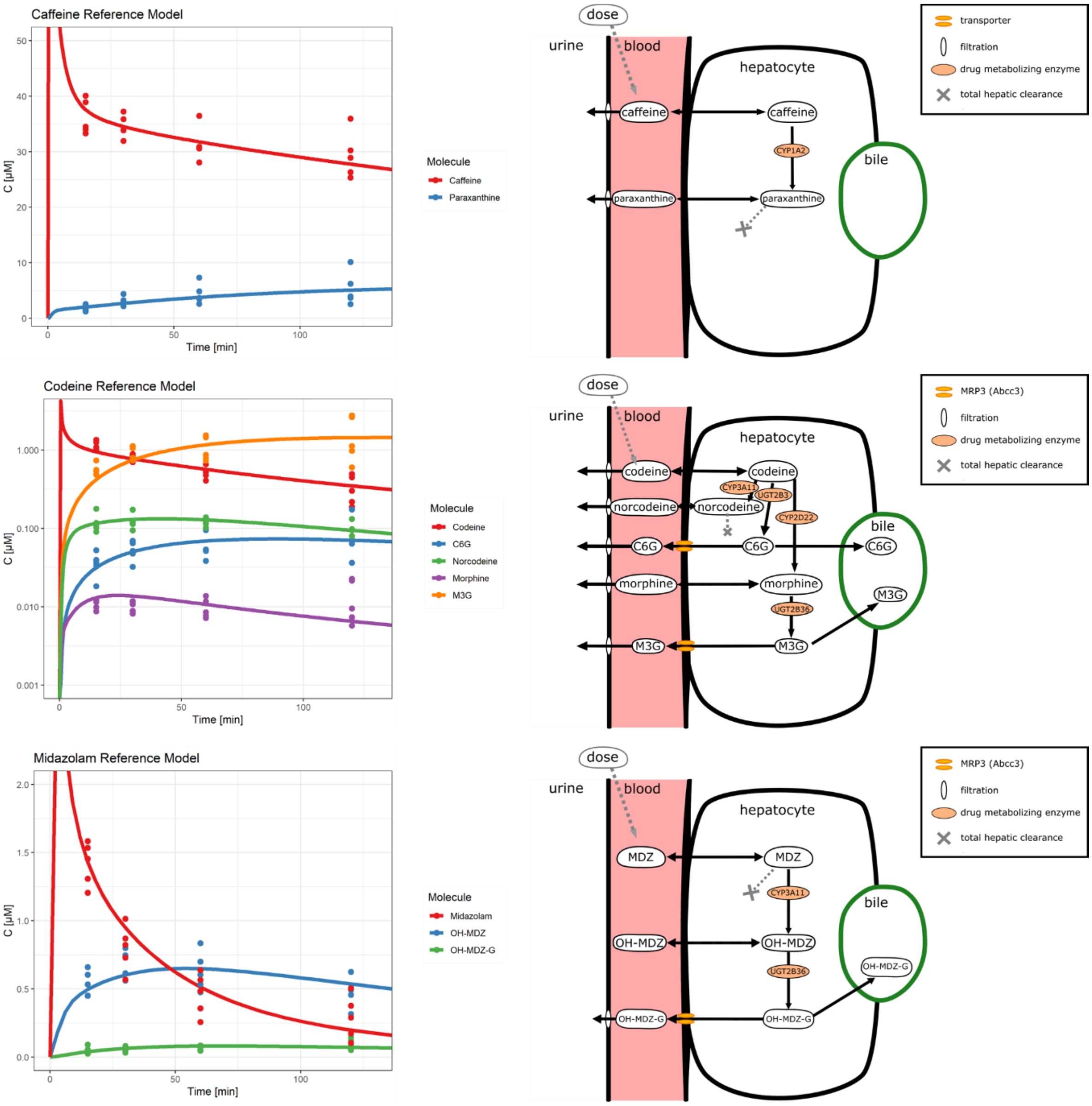

Figure S1: Reference PBPK models for caffeine, codeine, and midazolam. The left panels show the time courses of the plasma concentration of the parent drug and the metabolites (dots: measured concentration, lines: simulated concentration). The right panels show a schematic representation of the processes included in the reference PBPK models such as intravenous drug administration (dose), drug distribution either by diffusion or drug transport, drug metabolism by CYP enzymes, UGT enzymes, or total hepatic clearance, biliary clearance, and renal clearance. (A) Caffeine (B) Codeine (C) Midazolam. Abbreviations: PBPK, physiologically based pharmacokinetic; CYP, cytochrome

p450, UGT, UDP glucuronosyl transferase; C6G, codeine-6-glucuronide; M3G, morphine-3-glucuronide, OH-MDZ, 1-hydroxymidazolam, OH-MDZ-G, 1-hydroxymidazolam glucuronide.

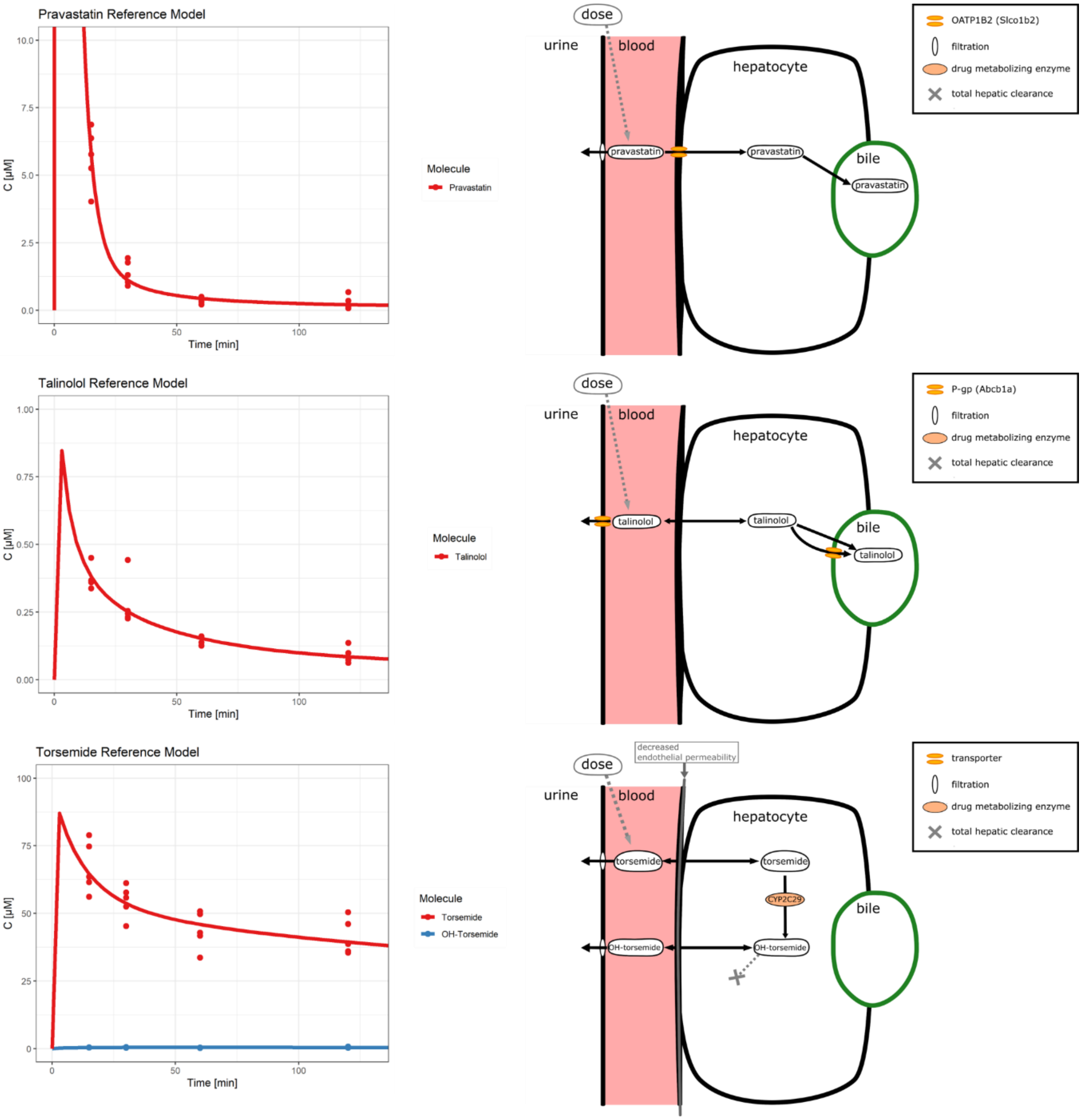

Figure S2: Reference PBPK models for pravastatin, talinolol, and torsemide. The left panels show the time courses of the plasma concentration of the parent drug and the metabolites (dots: measured concentration, lines: simulated concentration). The right panels show a schematic representation of the processes included in the reference PBPK models such as intravenous drug administration (dose), drug distribution either by diffusion or drug transport, drug metabolism by CYP enzymes or total hepatic clearance, biliary clearance, and renal clearance. (A) Pravastatin (B) Talinolol (C) Torsemide. Abbreviations: PBPK, physiologically based pharmacokinetic; CYP, cytochrome p450; OH-torsemide, hydroxytorsemide.

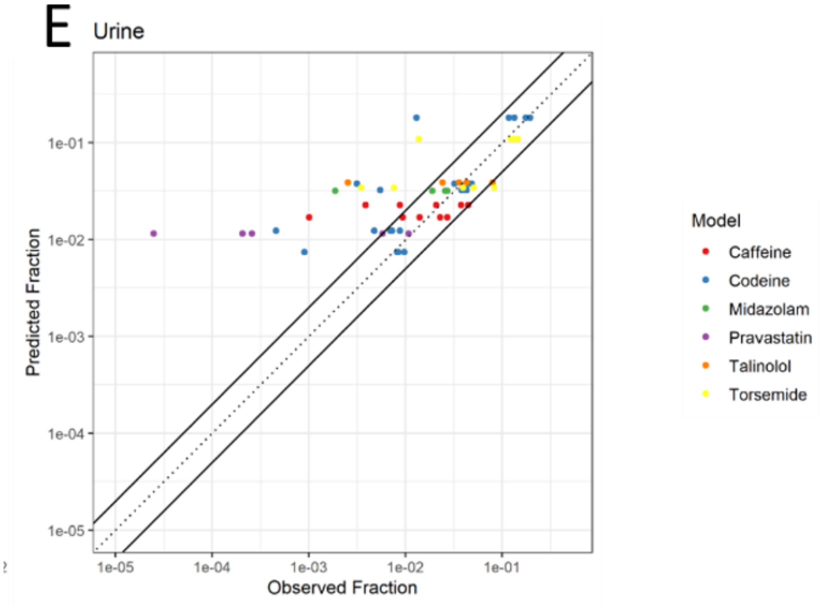

Figure S3: Predicted versus observed plot of fractions of dose in urine for all six PBPK models.

5. AUC in control and cirrhotic mice

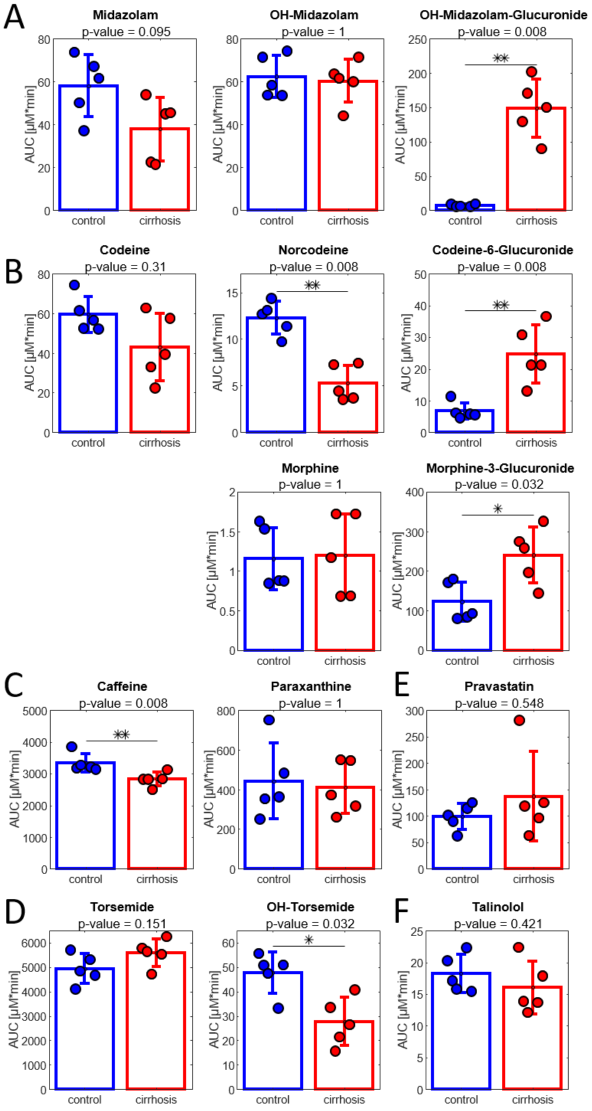

Figure S4: Bar plots of plasma AUCs (0-2 hours). Dots display the individual observed values, bars the mean value and the error bars the standard deviation. Column 1: Parent drugs; column 2: phase I metabolites; column 3: phase II metabolites (glucuronides). AUCs of the following molecules were significantly different between the groups (Wilcoxon test, two-sided, α=0.05): Caffeine, norcodeine, OH-torsemide, OH-midazolam-glucuronide, codeine-6-glucoronide, morphine-3-glucoronide. *: p<=0.05, **: p<=0.01. Abbreviations: AUC, Area under the concentration time curve.

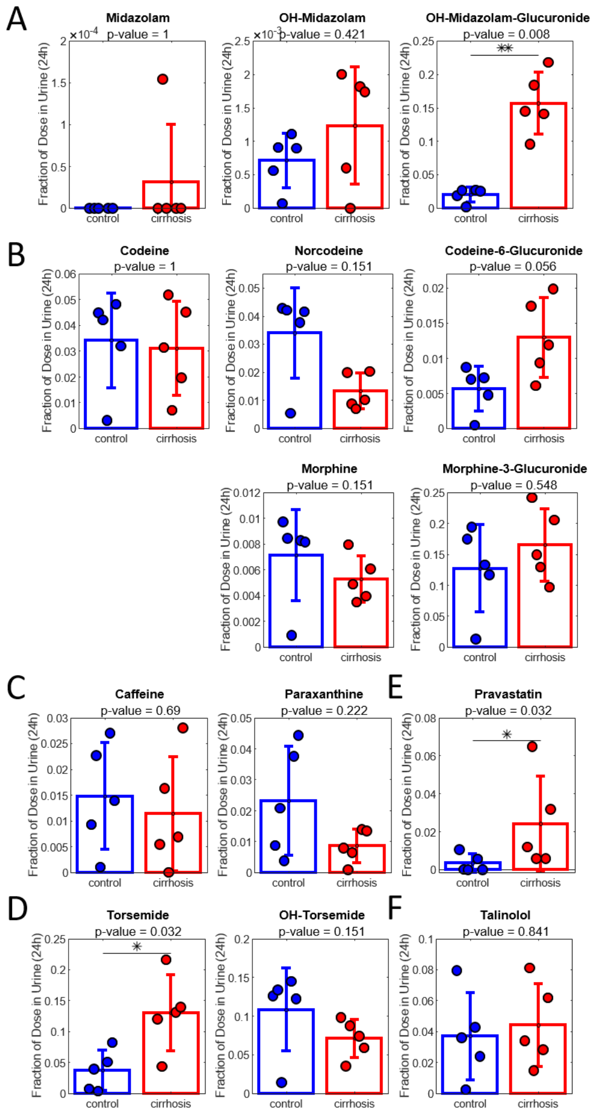

Figure S5: Bar plots of urine fractions (24 hours). Dots display the individual observed values, bars the mean value and the error bars the standard deviation. Column 1: Parent drugs; column 2: phase I metabolites; column 3: phase II metabolites (glucuronides). AUCs of the following molecules were significantly different between the groups (Wilcoxon test, two-sided, α=0.05): torsemide, pravastatin, morphine-3-glucorinide. *: p<=0.05, **: p<=0.01. Abbreviations: AUC, Area under the concentration time curve.

6. Hypothesis testing

Table S21: Parameters fitted for hypothesis testing.

	Codeine		Midazolam	
	**Coupled**	**Independent**	**Coupled**	**Independent**
**H1: Increased UGT activity**	Biliary Cl (M3G)<br>Biliary Cl (C6G)<br>v_{max} Abcc3 (M3G)<br>v_{max} Abcc3 (C6G)	UGT activity (M3G)<br>UGT activity (C6G)<br>norcodeine total hepatic Cl	Biliary Cl<br>(OH-MDZ-G)<br>v_{max} Abcc3 (OH-MDZ-G)	UGT activity<br>(OH-MDZ-G)
**H2: Impaired biliary clearance**	UGT activity (M3G)<br>UGT activity (C6G)<br>v_{max} Abcc3 (M3G)<br>v_{max} Abcc3 (C6G)	Biliary Cl (M3G)<br>Biliary Cl (C6G)<br>norcodeine total hepatic Cl	UGT activity (OH-MDZ-G)<br>v_{max} Abcc3 (OH-MDZ-G)	Biliary Cl<br>(OH-MDZ-G)
**H3: Increased sinusoidal transport**	UGT activity (M3G)<br>UGT activity (C6G)<br>Biliary Cl (M3G)<br>Biliary Cl (C6G)	Abcc3 reference concentration<br>norcodeine total hepatic Cl	Biliary Cl<br>(OH-MDZ-G)<br>UGT activity (OH-MDZ-G)	v_{max} Abcc3<br>(OH-MDZ-G)
Other optimized parameters<br>(coupled for all hypotheses)	logP (codeine)<br>Cyp3a11 activity (norcodeine)<br>Cyp2d22 activity (morphine)<br>GFR fraction (codeine, C6G, norcodeine, morphine, M3G)		total hepatic Cl (MDZ)<br>Cyp3a11 activity (OH-MDZ)<br>GFR fraction (OH-MDZ-G)<br>fu (OH-MDZ-G)	

For metabolic reactions, metabolites are mentioned in brackets after the enzyme name. Cl: Clearance

The goodness of fit was determined by inspection of visual predictive check plots, especially those of the metabolites OH-MDZ-G, C6G, M3G, and Norcodeine, and the root-mean-square deviation (RMSD, *Equation 1*).

Equation 1

$$RMSD = \sqrt{\frac{\sum_{i=1}^{n}(C_{pred,i} - C_{obs,i})^2}{n}}$$

Here, C_{pred} and C_{obs} designate the predicted and the observed drug concentration of the ith observation; n is the total number of observations.

Table S22: Results from PBPK hypothesis testing: Values of optimized parameters.

	Midazolam	Codeine	
	parameter ratio cirrhosis/control	parameter ratio cirrhosis /control	
	OH-MDZ-G	C6G	M3G
**H1: Increased UGT activity**			
UGT Vmax [1/min]	0.05	0.08	61.41
**H2: Impaired biliary clearance**			
Biliary Clearance Rate [1/min]	46.98	42.43	4.48
**H3: Increased sinusoidal transport** (Abcc3, v_{max}) [µmol/ml/min]	0.08	1.47	9.92

Abbreviations: H1-H3, hypothesis 1-3; CCl4, carbon tetrachloride; OH-MDZ-G, 1-hydroxymidazolam glucuronide; C6G, codeine-6-glucuronide; M3G, morphine-3-glucuronide; UGT, UDP glucuronosyl transferase, vmax, maximum velocity.

Table S23: Results from PBPK hypothesis testing: Ratios of optimized parameters.

	Midazolam	Codeine	
	parameter ratio cirrhosis/control	parameter ratio cirrhosis /control	
	**OH-MDZ-G**	**C6G**	**M3G**
**H1: Increased UGT activity**			
UGT Vmax	28.32	3.85	0.96
**H2: Impaired biliary clearance**			
Biliary Clearance Rate	$3.7*10^{-4}$	0.09	$1.2*10^{-8}$
**H3: Increased sinusoidal transport**			
(Abcc3, v_{max})	110.72	12.08	12.08

Abbreviations: H1-H3, hypothesis 1-3; CCl4, carbon tetrachloride; OH-MDZ-G, 1-hydroxymidazolam glucuronide; C6G, codeine-6-glucuronide; M3G, morphine-3-glucuronide; UGT, UDP glucuronosyl transferase, vmax, maximum velocity.

6.1. Midazolam

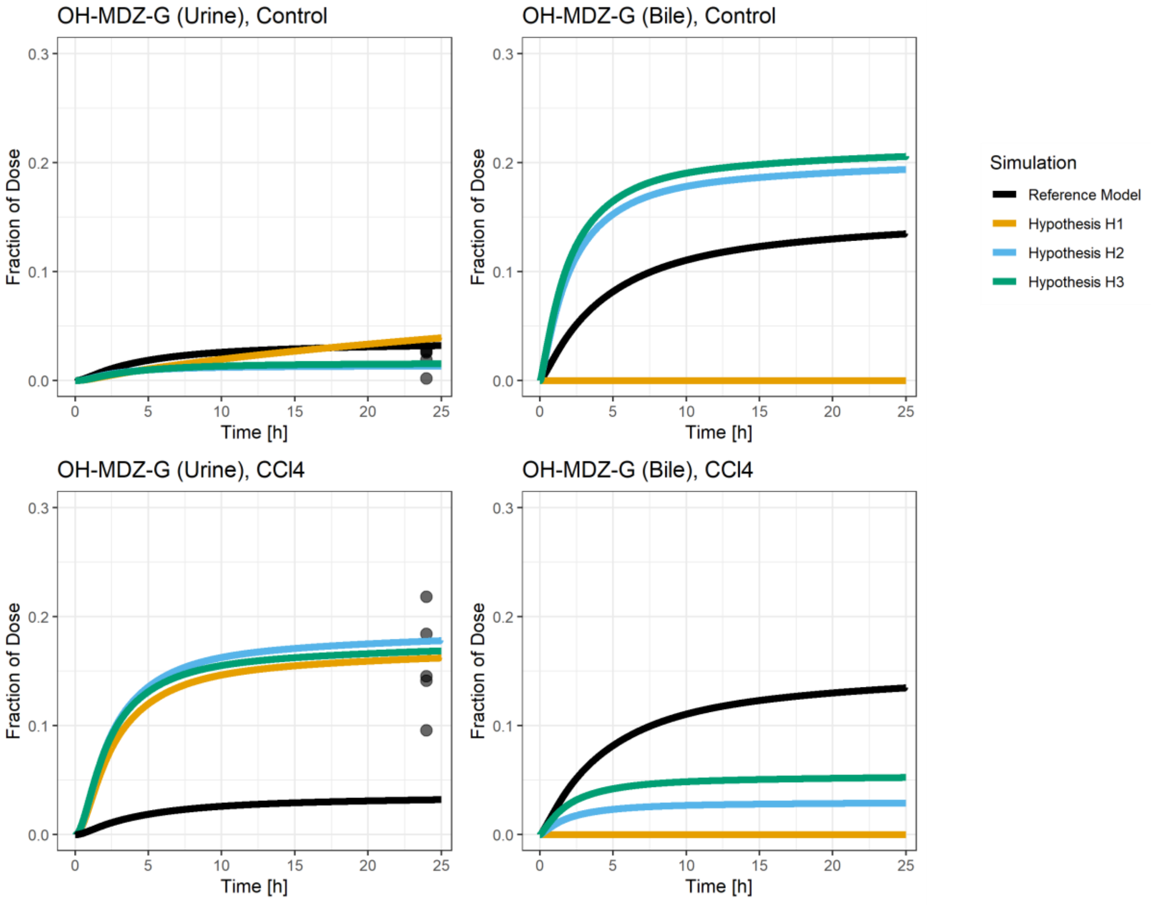

Figure S6: Midazolam PBPK simulations of the three hypotheses. Dots display observed fractions of doses, lines display the PBPK model simulations. The line color specifies the simulated PBPK model fit. The plots in the upper row show the control data and simulations, whereas the plots in the lower row show the cirrhosis data and simulations. Abbreviations: CCl4, carbon tetrachloride; OH-MDZ-G, 1-hydroxymidazolam glucuronide.

6.2. Codeine

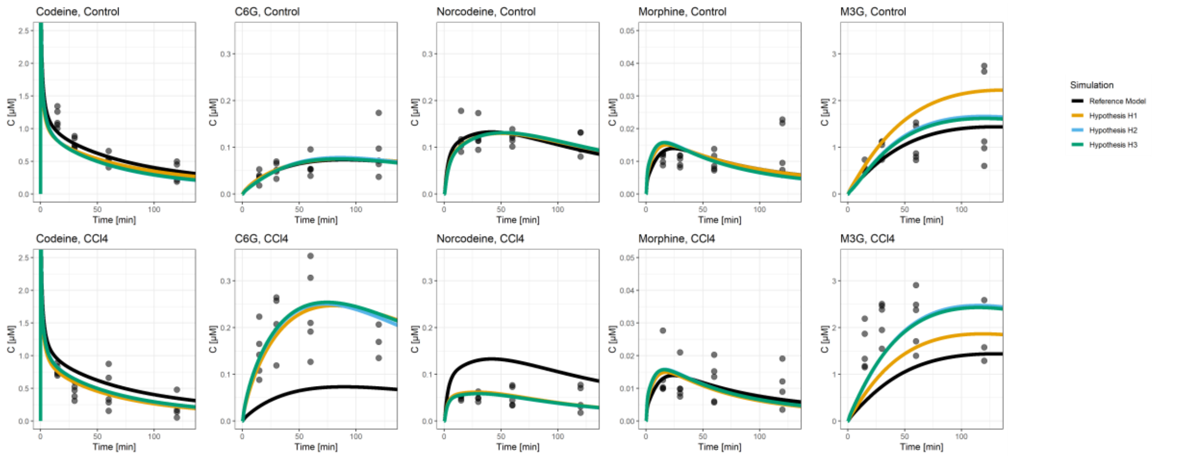

Figure S7: Codeine PBPK simulations of the three hypotheses. Dots display observed plasma concentrations, lines display the PBPK model simulations. The line color specifies the simulated PBPK model fit. The plots in the upper row show the control data and simulations, whereas the plots in the lower row show the cirrhosis data and simulations. Abbreviations: CCl4, carbon tetrachloride; C6G, codeine-6-glucuronide; M3G, morphine-3-glucuronide.

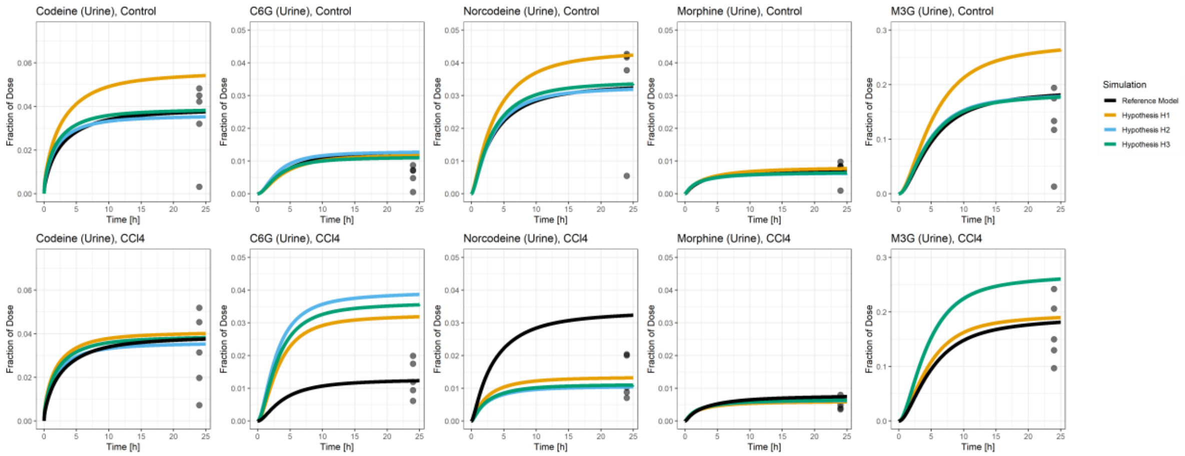

Figure S8: Codeine PBPK simulations of the three hypotheses. Dots display observed fractions of dose in urine, lines display the PBPK model simulations. The line color specifies the simulated PBPK model fit. The plots in the upper row show the control data and simulations, whereas the plots in the lower row show the cirrhosis data and simulations. Abbreviations: CCl4, carbon tetrachloride; C6G, codeine-6-glucuronide; M3G, morphine-3-glucuronide.

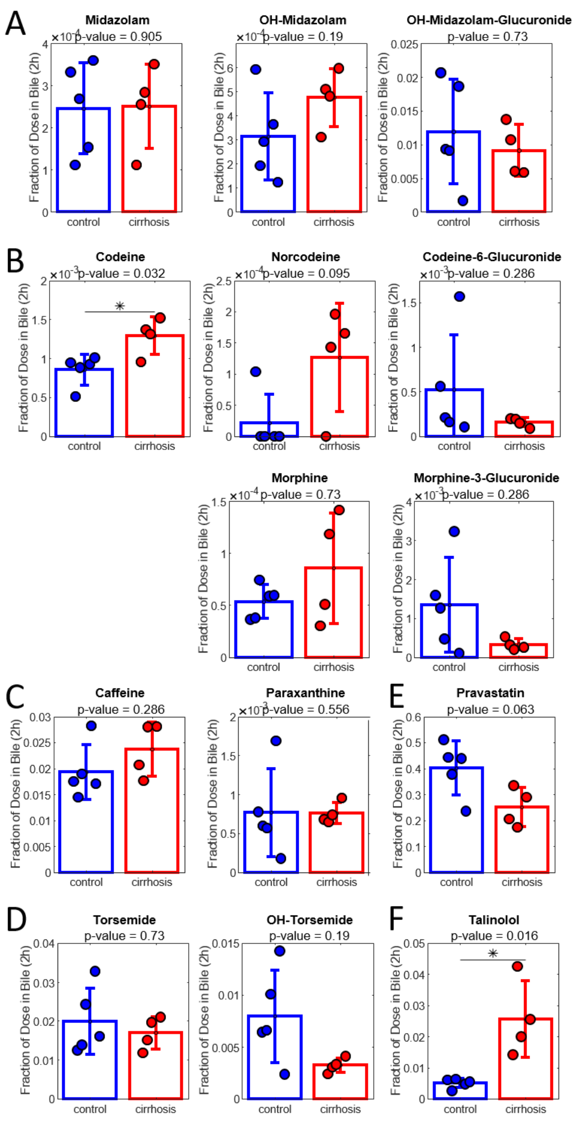

Figure S9: Bar plots of bile fractions (2 hours). Dots display the individual observed values, bars the mean value and the error bars the standard deviation. Column 1: Parent drugs; column 2: phase I metabolites; column 3: phase II metabolites (glucuronides). AUCs of the following molecules were significantly different between the groups (Wilcoxon test, two-sided, α=0.05): codeine, talinolol. *: p<=0.05, **: p<=0.01. Abbreviations: AUC, Area under the concentration time curve

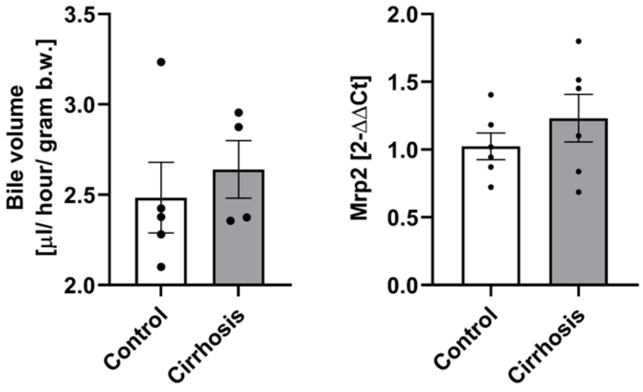

Figure S10: Bile flow rate (left) and RNA expression of the canalicular drug transporter Mrp2 (right).

7. In vitro CYP and UGT activity

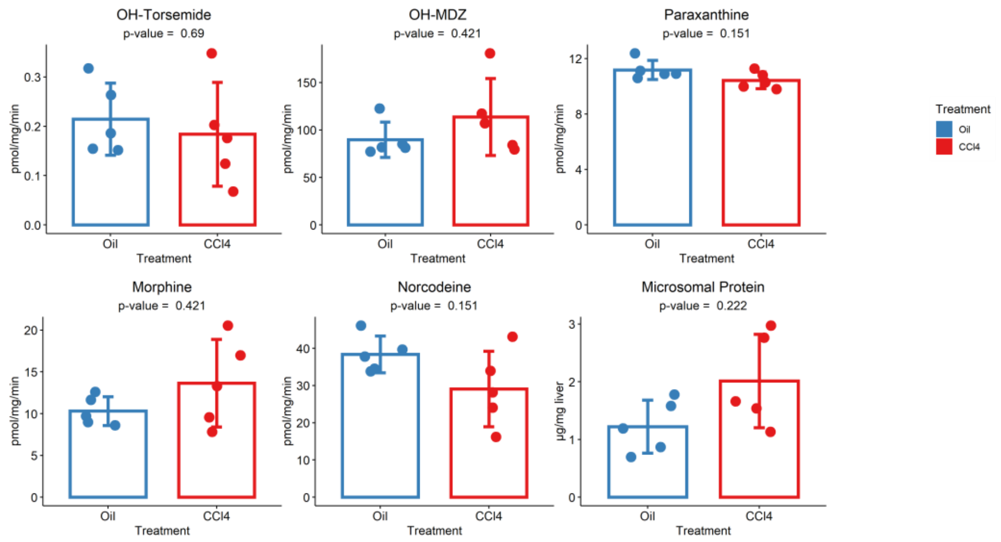

Figure S11: Bar plots of in vitro CYP activity assays in liver microsomes. Dots display the observed values, bars the mean value, and the error bars the standard deviation. P-values were calculated with two-sided Wilcoxon tests. The first five plots show metabolite formation rates in liver microsomes of OH-torsemide (p-value=0.69), OH-MDZ (p-value=0.421), paraxanthine (p-value 0.151), morphine (p-value=0.421), and norcodeine (p-value=0.151). The last plot shows the microsomal protein content of the liver tissue (p-value=0.222). Abbreviation: CCl4, carbon tetrachloride; OH-torsemide, hydroxytorsemide, OH-MDZ, 1-hydroxy midazolam

8. Formation of ascites, hepatic blood flow and plasma protein binding is of minor relevance

It is well known that cirrhosis may lead to ascites formation, altered blood flow and plasma protein binding which in turn may influence pharmacokinetics. Since it is possible to consider these pathophysiological parameters in PBPK models, we studied if they influence the pharmacokinetics of the here analyzed drug cocktail. Values from liver cirrhosis patients with pathophysiological conditions, such as ascites, altered blood flow and reduced plasma protein binding were translated to the mouse PBPK models to simulate mild, moderate, and severe liver cirrhosis (Table S21-23). Simulation of ascites had only a minor effect on drug cocktail PK and most AUC ratios were within the bioequivalence range (0.8-1.25) (Fig. S10). Altered blood flow led to an increase in the AUC of most parent drugs, whereas, the AUC of the metabolites decreased. Particularly, the AUCs of pravastatin and morphine were strongly changed in response to altered blood flow. Moreover, a decrease in plasma protein binding reduced the AUCs of the drugs and metabolites. However, it should be considered that no significant differences in albumin concentrations were observed between the cirrhotic and control mice (Fig. S11). Therefore, this mechanism – although relevant in patients – is unlikely to influence drug metabolism in the here-applied animal model. In conclusion, the simulated effects of the specific pathophysiological conditions were compound specific and cannot explain the above-described general changes in pharmacokinetics, such as the increased concentrations of glucuronides in the blood of cirrhotic mice.

Table S24: Parameter values for the simulation of ascites, altered blood flow, and increased fraction unbound (values taken from (Edgington and Willmann, 2008) except for ascites).

		Cirrhosis Stage		
		**mild**	**moderate**	**severe**
**Pathophysiological change**		**(CP-A)**	**(CP-B)**	**(CP-C)**
**Ascites**		5 ml	7.5 ml	10 ml
**Blood flows***	Portal vein	0.40	0.36	0.04
	Hepatic artery	1.3	2.3	3.4
	Kidney	0.88	0.65	0.48
	Cardiac output	1.11	1.27	1.36
**fu (albumin)***		0.80	0.68	0.50

* fraction of control

8.1 Simulation of impaired hepatic metabolism

To simulate impaired hepatic CYP activity, the *CLspec/[Enzyme] interaction factor* in liver cells and all *total hepatic clearances* that represented a CYP reaction were rescaled. The UGT enzyme activities and extrahepatic metabolism were not modified. The first predictions were based on the reduction of the CYP1A expression area, thus the parameter values were set to 10.85% of their reference value.

8.2 Simulation of altered transporter expression

To simulate the effect of altered transporter expression in the liver, the normalized relative expression in the cellular compartment of the liver was scaled according to the fold change observed in the RNA-seq experiment.

8.3 Simulation of altered blood flows

Blood flows (Q) of the mouse PBPK model were scaled based on previously published values for patients(Edginton and Willmann, 2008) (Table S22). In PK-Sim PBPK models, the liver blood flow only accounts for the blood flow in the hepatic artery, whereas the portal vein blood flow is defined separately as the sum of the blood flows from all gastrointestinal organs. To achieve the target blood flow in the portal vein, all gastrointestinal organ blood flows were multiplied with the respective factor (Table S23).

The lung blood flow equals the cardiac output (CO) because all blood from the systemic circulation passes the lung. In PK-Sim, the lung blood flow is also defined as a formula like the portal vein blood flow. To achieve the target blood flows in the portal vein and the lung, the blood flows of the organs draining into the portal vein and the lung were changed according to *Equation 2*-*Equation 4*.

Equation 2

$$CO_{remainder} = CO_{healthy} - Q_{liver,healthy} - Q_{portal\ vein} - Q_{kidney}$$

Equation 3

$$CO_{target,remainder} = CO_{healthy} * relativeChangeCO - Q_{liver,healthy} * relativeChangeHepaticArtery - Q_{portal\ vein} * relativeChangePortalVein - Q_{kidney} * relativeChangeKidney$$

Equation 4

$$scalingFactor = \frac{CO_{target,remainder}}{CO_{remainder}}$$

Table S25: Scaling factors of PBPK model blood flows to according to Child-Pugh (CP) class (Edginton and Willmann, 2008)

	**Organ blood flow**	**Child-Pugh Class**		
		CP-A	CP-B	CP-C
**Cardiac Output**	lung	1.11	1.27	1.36
**Portal Vein**	stomach<br>small intestine<br>large intestine<br>pancreas<br>spleen	0.40	0.36	0.04
**Hepatic Artery**	liver	1.3	2.3	3.4
**Kidney**	kidney	0.88	0.65	0.48
**Scaling factor***	muscle<br>fat<br>skin<br>bone<br>brain<br>heart<br>gonads	1.82	2.25	2.68

*Scaling factor for the remaining organ blood flows to achieve the target blood flows in lung, hepatic artery, portal vein and kidney

8.4 Simulation of ascites

Based on physical examination of the CCl_4 mice, the maximum volume (V) of ascites was estimated to about 10 ml which corresponds to one third of the mice's body weight (oral communication by Ahmed Ghallab). Ascites designates increased fluid in the body cavity and thus an increased interstitial volume. In the cirrhosis ($_{cirr}$) PBPK models, ascites was simulated by increasing the interstitial volume of the large intestine by 5 ml, 7.5 ml, or 10 ml ($V_{ascites}$). The smaller ascites volumes simulate mild and moderate ascites.

In PK-Sim, the large intestine's intracellular and vascular volume are formulas, which are defined as a certain fraction of the total organ volume. If the organ volume increases due to ascites, the intracellular and the vascular volume will change accordingly. To keep intracellular and vascular volumes in the large intestine equal to the reference model volumes because they are not supposed to change with ascites, the interstitial, intracellular and vascular fractions in the large intestine were rescaled (*Equation 5* - *Equation 10*).

The total organ blood flow is also defined as a formula in PK-Sim. The specific blood flow rate (Q_{spec}) is multiplied by the organ volume. The specific blood flow rate was rescaled so that the blood flow through the large intestine equals the reference model blood flow rate (*Equation 11*-*Equation 12*).

Equation 5

$$V_{vascular,healthy} = Fraction_{vascular,healthy} * V_{large\ intestine,healthy}$$

Equation 6

$$V_{intracellular,healthy} = Fraction_{intracellular,cirr} * V_{large\ intestine,healthy}$$

Equation 7

$$V_{cirr} = V_{large\ intestine,healthy} + V_{ascites}$$

Equation 8

$$Fraction_{vascular,cirr} = \frac{V_{vascular,healthy}}{V_{cirr}}$$

Equation 9

$$Fraction_{intracellular,cirr} = \frac{V_{intracellular,healthy}}{V_{cirr}}$$

Equation 10

$$Fraction_{interstitial,cirr} = 1 - Fraction_{intracellular,cirr} - Fraction_{vascular,cirr}$$

Equation 11

$$relativeBloodFlowChange = \frac{V_{large\ intestine,healthy}}{V_{cirr}}$$

Equation 12

$$Q_{spec,interstitial,cirr} = Q_{spec,interstitial} * relativeBloodFlowChange$$

8.5 Simulation of reduced plasma protein concentrations

To simulate the impact of cirrhosis ($_{cirr}$) on plasma protein binding, the fractions unbound were recalculated for each molecule individually according to Equation 13 (Edginton and Willmann, 2008). Based on the fraction unbound (fu) in the reference model and the plasma protein volume fraction in healthy individuals ($f_{protein}$=0.0224), the protein/plasma partition coefficient (K_{prot}) was calculated (*Equation 14*). K_{prot} is a measure of the drug's affinity to plasma proteins and remains constant because it is not affected by reduced concentrations of plasma proteins. For the calculation of the altered fractions unbound (*Equation 15*), the volume fraction of the plasma proteins ($f_{protein}$) was scaled to 80%, 68%, and 50% of the initial value(Edginton and Willmann, 2008). Although $f_{protein}$ is a human value and the mouse $f_{protein}$ is probably different, the calculated fu_{cirr} is only dependent on the relative change of $f_{protein}$. However $K_{protein}$ depends on $f_{protein}$. Thus, $K_{protein}$ is probably not the true $K_{protein}$ for mouse but only an apparent parameter for calculation.

Equation 13

$$fu = \frac{1}{\left(1 - f_{protein,healthy}\right) + f_{protein,healthy} * K_{protein}}$$

Equation 14

$$K_{protein} = \frac{\frac{1}{fu} - (1 - f_{protein,healthy})}{f_{protein,healthy}}$$

Equation 15

$$fu_{cirr} = \frac{1}{\left(1 - f_{protein,cirr}\right) + f_{protein,cirr} * K_{protein}}$$

Table S26 Scaled fractions unbound used in the simulations of liver cirrhosis pathophysiology

	**fraction unbound**			
**Molecule**	**Control**	**CP-A**	**CP-B**	**CP-C**
Caffeine	79.1%	82.6%	84.8%	88.3%
Paraxanthine	85.0%	87.6%	89.3%	91.9%
Codeine	75.0%	78.9%	81.5%	85.7%
C6G	100.0%	100.0%	100.0%	100.0%
Norcodeine	76.5%	80.3%	82.7%	86.7%
Morphine	75.0%	78.9%	81.5%	85.7%
M3G	90.0%	91.8%	93.0%	94.7%
Midazolam	8.2%	10.0%	11.6%	15.2%
OH-MDZ	15.0%	18.1%	20.6%	26.1%
OH-MDZ-G	60.2%	65.4%	68.9%	75.1%
Pravastatin	67.2%	71.9%	75.1%	80.4%
Talinolol	39.0%	44.4%	48.5%	56.1%
Torsemide	0.1%	0.1%	0.1%	0.2%
OH-torsemide	5.9%	7.3%	8.4%	11.1%

C6G, codeine-6-glucuronide; M3G, morphine-3-glucuronide; OH-MDZ, 1-hydroxymidazolam; OH-MDZ-G, 1-hydroxymidazolam glucuronide; CP,Child-Pugh.

8.6 *Simulation of liver cirrhosis pathophysiology*

Pathophysiologic changes were simulated with R (R Core Team, 2020) and the R toolbox of the OSP Suite. The parameterization of the reference models was adjusted to account for reduced drug-metabolizing enzyme expression, altered transporter expression, ascites, altered liver blood flow, or reduced plasma protein concentration.

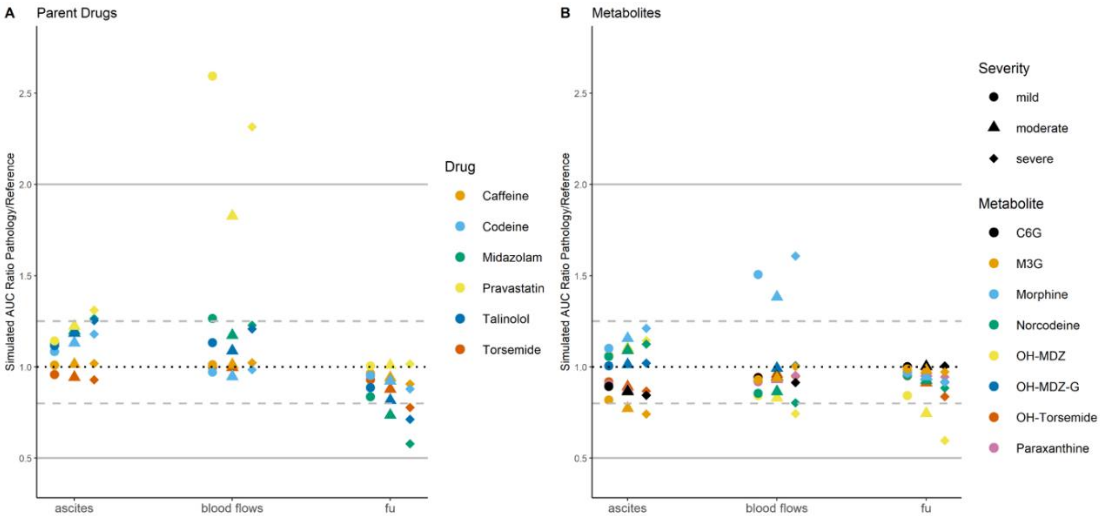

Figure S12: Simulated AUC ratios. (A+B) Simulated AUC ratios for different pathophysiologic changes associated with liver cirrhosis. The impact of ascites, altered blood flows and increased fractions unbound (fu) were simulated with parameter values corresponding to mild, moderate, and severe cirrhosis(Edginton and Willmann, 2008). The simulation time for the calculation of the AUC was two hours. The simulated molecule is indicated by the color, the severity is indicated by the shape. The bioequivalence range (0.8-1.25) is marked by dashed grey lines, the twofold range is marked by the solid grey lines. (A) parent drugs, (B) metabolites.

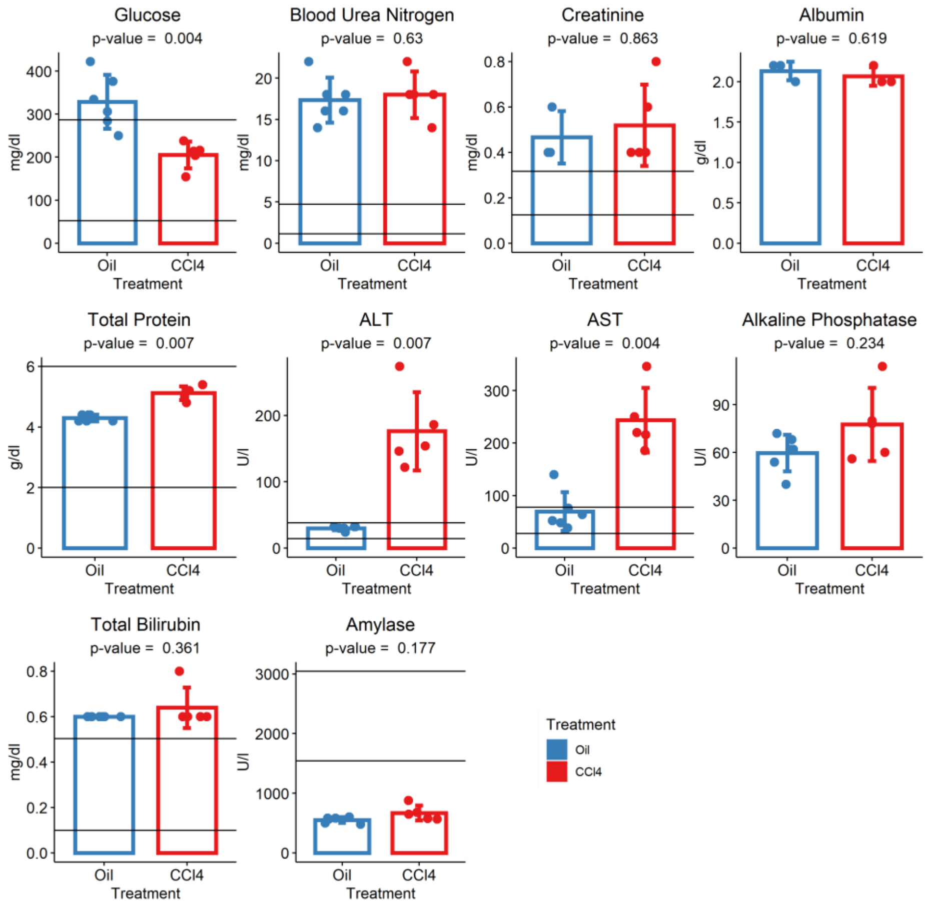

Figure S13: Bar plots of liver function parameters. Dots display the observed values, bars the mean value, and the error bars the standard deviation. Horizontal black lines indicate reference ranges for 10-12 week old C57/BL6 mice (Boehm et al., 2007). P-values were calculated with two-sided Wilcoxon tests. Abbreviations: CCl4, carbon tetrachloride; AST, aspartate transaminase; ALT, alanine transaminase.

9. Changes in transporter gene expression

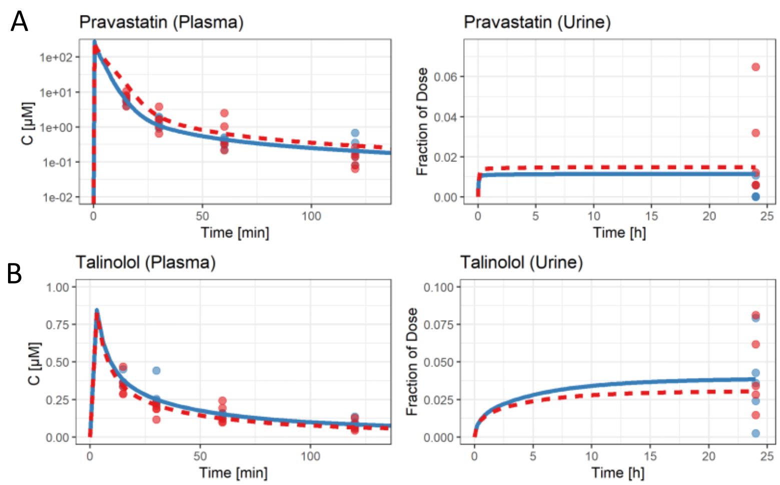

Figure S14: Simulated effect of altered transporter expression on drug cocktail disposition for pravastatin (A) and talinolol (B). Dots show the observed plasma concentrations, lines show the simulated plasma concentrations. Blue indicates the control group and the reference model simulation. Red indicates the cirrhosis mice. Transporter expression in the liver was scaled according to the fold change observed from RNA-seq data.

10. Bioinformatics of transcriptomics data sets

The chronic CCl_4 mouse model has been published in (Holland et al., 2022) and the data were uploaded in the corresponding publicly available Zenodo archive (10.5281/zenodo.7242764). The raw data are also available at Gene Expression Omnibus (GEO) under accession ID GSE167216. The transcriptomics data of the patients with chronic liver disease obtained from (Holland et al., 2022). These data were downloaded from the Zenodo archive (10.5281/zenodo.7242764). For the analysis of human chronic liver disease patients with fibrosis stage 6 reported in (Hoang et al., 2019) were compared to stage 0 as a reference.

All bioinformatics related analyses were performed in R (version 4.2.1). The source code is publicly available on Github (https://github.com/christianholland/cirrhosis-metabolism). If not stated otherwise a mouse gene was considered differentially expressed if $|logFC| >= 1$ and $FDR <= 0.05$. A human gene was classified as differentially expressed with a more relaxed cutoff of $|logFC| >= log2(1.5)$ and $FDR <= 0.2$. The correlation analysis was performed using the log-fold change (logFC) of human and mouse genes. Therefore, it was required that the genes symbols of both species are in the same name space. Hence, we translated the human HGNC symbols to their ortholog MGI symbols using the R/Bioconductor package biomart (version 2.44.0), which itself queries the Ensembl Archive Release 99. For the case that many HGNC symbols were translated to the same MGI symbol the logFC of the resulting mouse gene was the arithmetic mean of the logFC of the human genes. The result of the correlation analysis was summarized as the Pearson correlation coefficient *r*.

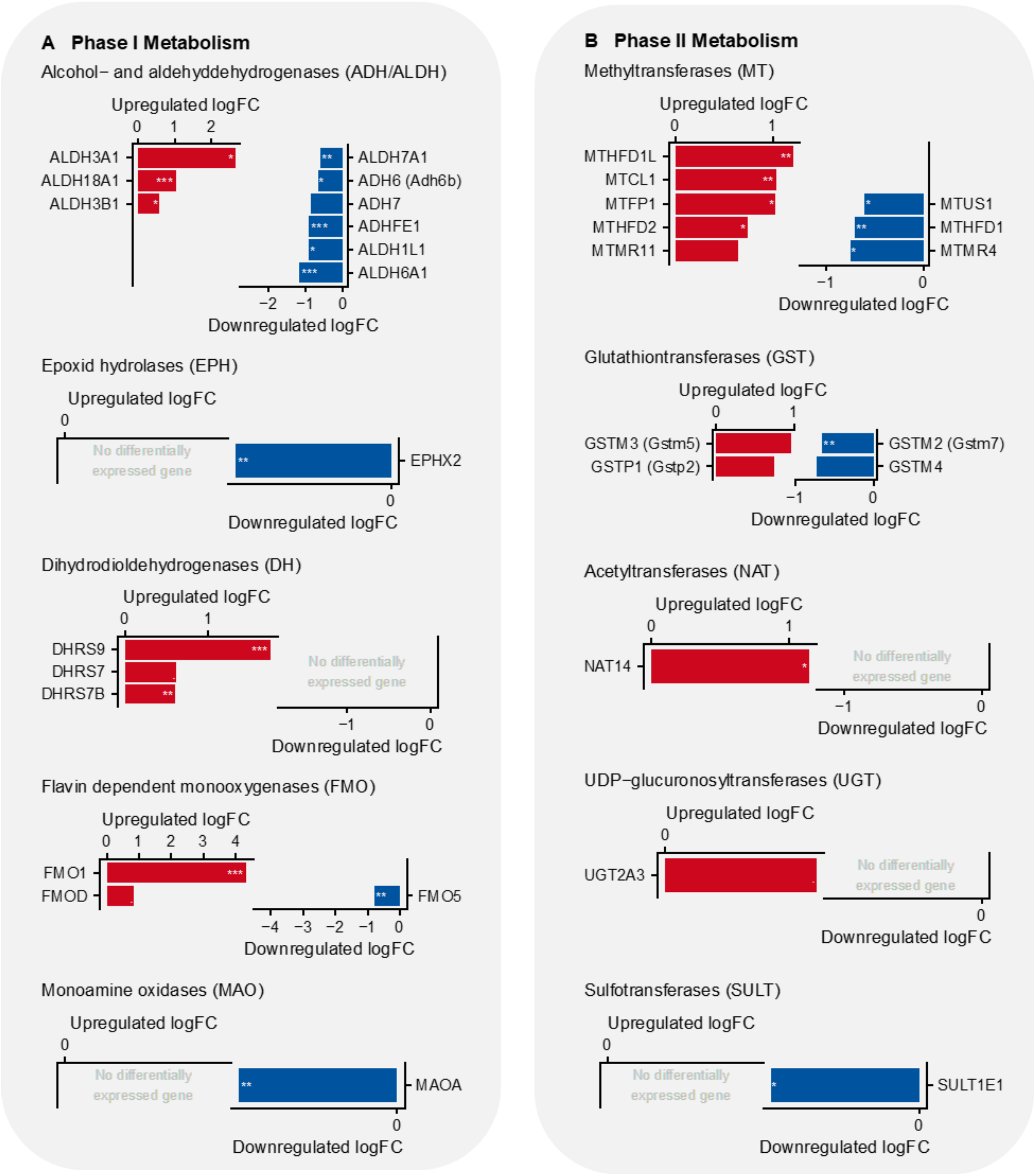
A Phase I Metabolism
Alcohol− and aldehyddehydrogenases (ADH/ALDH)
Upregulated logFC
Downregulated logFC
ALDH3A1
ALDH18A1
ALDH3B1
ALDH7A1
ADH6 (Adh6b)
ADH7
ADHFE1
ALDH1L1
ALDH6A1
Epoxid hydrolases (EPH)
No differentially expressed gene
EPHX2
Dihydrodioldehydrogenases (DH)
DHRS9
DHRS7
DHRS7B
Flavin dependent monooxygenases (FMO)
FMO1
FMOD
FMO5
Monoamine oxidases (MAO)
MAOA
B Phase II Metabolism
Methyltransferases (MT)
MTHFD1L
MTCL1
MTFP1
MTHFD2
MTMR11
MTUS1
MTHFD1
MTMR4
Glutathiontransferases (GST)
GSTM3 (Gstm5)
GSTP1 (Gstp2)
GSTM2 (Gstm7)
GSTM4
Acetyltransferases (NAT)
NAT14
UDP−glucuronosyltransferases (UGT)
UGT2A3
Sulfotransferases (SULT)
SULT1E1

Figure S15

11. Gene zonation

Based on a single cell RNA-seq atlas of the mouse liver (19) we differentiated preferentially pericentrally versus uniformly expressed metabolizing enzymes (Fig S16). Indeed, expression of *Cyp2e1*, *Cyp1a2* and *Cyp2c29* is 135-, 29-, and 50-fold higher in the most pericentral than in the most periportal layer of hepatocytes, respectively. In contrast, *Cyp3a11*, *Cyp2d22* and several further enzymes involved into the metabolism of the here investigated drugs such as Ugt2b36 are more uniformly expressed with pericentral-to-periportal expression ratios of less than five. In conclusion, decreased RNA expression was preferentially observed for enzymes with a pericentral expression pattern.

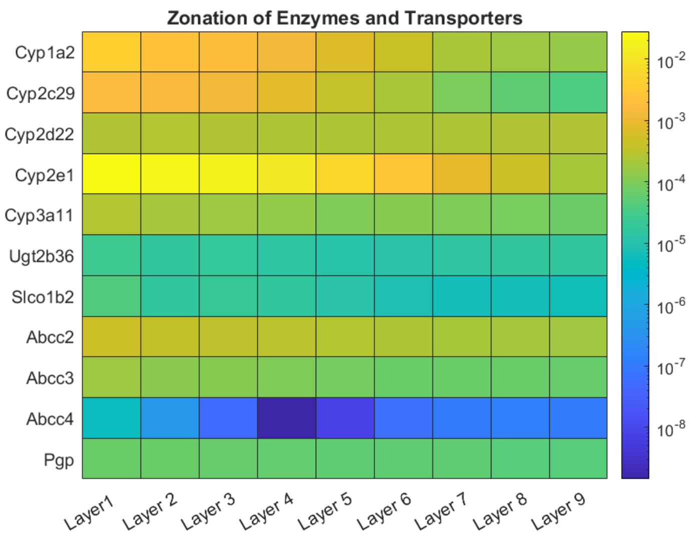

Figure S16: ADME genes zonation profiles. Heatmap shows ADME gene expression across different zones in livers of healthy C57BL/6 mice. Data were retrieved from Halpern et al. (2017)(Halpern et al., 2017). Layer 1 is the most pericentral hepatocyte layer, Layer 9 is the most periportal hepatocyte layer. The colour was scaled across the expression of all genes and layers. Yellow indicates the highest absolute gene expression, dark blue indicates the lowest absolute gene expression. The unit of gene expression was the fraction of total unique molecular identifiers. Abbreviations: AUC, Area under the concentration-time curve (0-2hours), fu, fraction unbound, C6G, codeine-6-glucuronide, M3G, morphine-3-glucuronide, OH-MDZ, 1-hydroxymidazolam, OH-MDZ-G, 1-hydroxymidazolam glucuronide, OH-torsemide, hydroxytorsemide.

References

Avdeef, A. (1996). Octanol-, Chloroform-, and Propylene Glycol Dipelargonat-Water Partitioning of Morphine-6-glucuronide and Other Related Opiates. *J. Med. Chem.* 39**,** 4377-4381.

Blanchard, J. (1982). Protein binding of caffeine in young and elderly males. *J Pharm Sci* 71**,** 1415-1418.

Boehm, O., Zur, B., Koch, A., Tran, N., Freyenhagen, R., Hartmann, M., and Zacharowski, K. (2007). Clinical chemistry reference database for Wistar rats and C57/BL6 mice. *Biol Chem* 388**,** 547-554.

Bonati, M., Latini, R., Tognoni, G., Young, J.F., and Garattini, S. (1985). Interspecies Comparison of In Vivo Caffeine Pharmacokinetics in Man, Monkey, Rabbit, Rat, and Mouse. *Drug Metabolism Reviews* 15**,** 1355-1383.

Bortolotti, A., Jiritano, L., and Bonati, M. (1985). Pharmacokinetics of paraxanthine, one of the primary metabolites of caffeine, in the rat. *Drug Metabolism and Disposition* 13**,** 227-231.

Britz, H., Hanke, N., Volz, A.K., Spigset, O., Schwab, M., Eissing, T., Wendl, T., Frechen, S., and Lehr, T. (2019). Physiologically-Based Pharmacokinetic Models for CYP1A2 Drug-Drug Interaction Prediction: A Modeling Network of Fluvoxamine, Theophylline, Caffeine, Rifampicin, and Midazolam. *CPT Pharmacometrics Syst Pharmacol* 8**,** 296-307.

Buters, J.T.M., Tang, B.-K., Pineau, T., Gelboin, H.V., Kimura, S., and Gonzalez, F.J. (1996). Role of CYP1A2 in caffeine pharmacokinetics and metabolism: studies using mice deficient in CYP1A2. *Pharmacogenetics* 6**,** 291-296.

Collett, A., Tanianis-Hughes, J., Hallifax, D., and Warhurst, G. (2004). Predicting P-Glycoprotein Effects on Oral Absorption: Correlation of Transport in Caco-2 with Drug Pharmacokinetics in Wild-Type and mdr1a(-/-) Mice in Vivo. *Pharm Res* 21**,** 819-826.

Edginton, A., Schmitt, W., and Willmann, S. (2006). Development and Evaluation of a Generic Physiologically Based Pharmacokinetic Model for Children. *Clin Pharmacokinet* 45**,** 1013-1034.

Edginton, A.N., and Willmann, S. (2008). Physiology-based simulations of a pathological condition: prediction of pharmacokinetics in patients with liver cirrhosis. *Clin Pharmacokinet* 47**,** 743-752.

Evans, A.M., O'brien, J.A., and Nation, R.L. (1999). Application of a Loading Wash-out Method for Inestigating the Hepatocellular Efflux of a Hepatically-generated Metabolite, Morphine-3-glucuronide. *J. Pharm. Pharmacol.* 51**,** 1289-1297.

Everett, D.W., Chando, T.J., Didonato, G.C., Singhvi, S.M., Pan, H.Y., and Weinstein, S.H. (1991). Biotransformation of pravastatin sodium in humans. *Drug Metab Dispos* 19**,** 740-748.

Fda (2017). *Torsemide Tablets Prescribing Information* [Online]. U.S. Food and Drug Administration (FDA). Available: https://www.accessdata.fda.gov/drugsatfda_docs/label/2017/020136s027lbl.pdf [Accessed 2022-02-21].

Fda (2020). *Pravastatin Sodium Tablets* [Online]. U.S. Food and Drug Administration (FDA). Available: https://www.accessdata.fda.gov/drugsatfda_docs/label/2020/019898s069lbl.pdf [Accessed 2022-02-21].

Fukuda, H., Ohashi, R., Tsuda-Tsukimoto, M., and Tamai, I. (2008). Effect of plasma protein binding on in vitro-in vivo correlation of biliary excretion of drugs evaluated by sandwich-cultured rat hepatocytes. *Drug Metab Dispos* 36**,** 1275-1282.

Gramatté, T., Oertel, R., Terhaag, B., and Kirch, W. (1996). Direct demonstration of small intestinal secretion and site-dependent absorption of the beta-blocker talinolol in humans. *Clinical Pharmacology & Therapeutics* 59**,** 541-549.

Halpern, K.B., Shenhav, R., Matcovitch-Natan, O., Tóth, B., Lemze, D., Golan, M., Massasa, E.E., Baydatch, S., Landen, S., Moor, A.E., Brandis, A., Giladi, A., Stokar-Avihail, A., David, E., Amit, I., and Itzkovitz, S. (2017). Single-cell spatial reconstruction reveals global division of labour in the mammalian liver. *Nature* 542**,** 352-356.

Hart, S.N., Cui, Y., Klaassen, C.D., and Zhong, X.B. (2008). Three Patterns of Cytochrome P450 Gene Expression during Liver Maturation in Mice. *Drug Metabolism and Disposition* 37**,** 116-121.

Heikkinen, A.T., Baneyx, G., Caruso, A., and Parrott, N. (2012). Application of PBPK modeling to predict human intestinal metabolism of CYP3A substrates - an evaluation and case study using GastroPlus. *Eur J Pharm Sci* 47**,** 375-386.
Hersman, E.M., and Bumpus, N.N. (2014). A targeted proteomics approach for profiling murine cytochrome P450 expression. *J Pharmacol Exp Ther* 349**,** 221-228.
Hoang, S.A., Oseini, A., Feaver, R.E., Cole, B.K., Asgharpour, A., Vincent, R., Siddiqui, M., Lawson, M.J., Day, N.C., Taylor, J.M., Wamhoff, B.R., Mirshahi, F., Contos, M.J., Idowu, M., and Sanyal, A.J. (2019). Gene Expression Predicts Histological Severity and Reveals Distinct Molecular Profiles of Nonalcoholic Fatty Liver Disease. *Sci Rep* 9**,** 12541.
Holland, C.H., Ramirez Flores, R.O., Myllys, M., Hassan, R., Edlund, K., Hofmann, U., Marchan, R., Cadenas, C., Reinders, J., Hoehme, S., Seddek, A.L., Dooley, S., Keitel, V., Godoy, P., Begher-Tibbe, B., Trautwein, C., Rupp, C., Mueller, S., Longerich, T., Hengstler, J.G., Saez-Rodriguez, J., and Ghallab, A. (2022). Transcriptomic Cross-Species Analysis of Chronic Liver Disease Reveals Consistent Regulation Between Humans and Mice. *Hepatol Commun* 6**,** 161-177.
Järvinen, E., Troberg, J., Kidron, H., and Finel, M. (2017). Selectivity in the Efflux of Glucuronides by Human Transporters: MRP4 Is Highly Active toward 4-Methylumbelliferone and 1-Naphthol Glucuronides, while MRP3 Exhibits Stereoselective Propranolol Glucuronide Transport. *Molecular Pharmaceutics* 14**,** 3299-3311.
Knauf, H., and Mutschler, E. (1998). Clinical Pharmacokinetics and Pharmacodynamics of Torasemide. *Clin Pharmacokinet* 34**,** 1-24.
Krauss, M., Hofmann, U., Schafmayer, C., Igel, S., Schlender, J., Mueller, C., Brosch, M., Von Schoenfels, W., Erhart, W., Schuppert, A., Block, M., Schaeffeler, E., Boehmer, G., Goerlitz, L., Hoecker, J., Lippert, J., Kerb, R., Hampe, J., Kuepfer, L., and Schwab, M. (2017). Translational learning from clinical studies predicts drug pharmacokinetics across patient populations. *npj Systems Biology and Applications* 3.
Kurita, A., Miyauchi, Y., Ikushiro, S., Mackenzie, P.I., Yamada, H., and Ishii, Y. (2017). Comprehensive Characterization of Mouse UDP-Glucuronosyltransferase (Ugt) Belonging to the Ugt2b Subfamily: Identification of Ugt2b36 as the Predominant Isoform Involved in Morphine Glucuronidation. *J Pharmacol Exp Ther* 361**,** 199-208.
Labedziki, A., Buters, J.T.M., Jabrane, W., and Fuhr, U. (2002). Differences in caffeine and paraxanthine metabolism between human and murine CYP1A2. *Biochemical Pharmacology* 63**,** 2159-2167.
Lee, D.Y., Kim, J.Y., Kim, Y.C., Kwon, J.W., Kim, W.B., and Lee, M.G. (2005). Dose-independent pharmacokinetics of torasemide after intravenous and oral administration to rats. *Biopharm Drug Dispos* 26**,** 173-182.
Lelo, A., Birkett, D.J., Robson, R.A., and Miners, J.O. (1986). Comparative pharmacokinetics of caffeine and its primary demethylated metabolites paraxanthine, theobromine and theophylline in man. *Br. J. clin. Pharmac.* 22**,** 177-182.
Lippert, J., Brosch, M., Von Kampen, O., Meyer, M., Siegmund, H.U., Schafmayer, C., Becker, T., Laffert, B., Gorlitz, L., Schreiber, S., Neuvonen, P.J., Niemi, M., Hampe, J., and Kuepfer, L. (2012). A mechanistic, model-based approach to safety assessment in clinical development. *CPT Pharmacometrics Syst Pharmacol* 1**,** e13.
Martignoni, M. (2006). *Species and strain differences in drug metabolism in liver and intestine.* Doctor of Philosophy, University of Groningen.
Martignoni, M., Groothuis, G.M., and De Kanter, R. (2006). Species differences between mouse, rat, dog, monkey and human CYP-mediated drug metabolism, inhibition and induction. *Expert Opin Drug Metab Toxicol* 2**,** 875-894.
Matthaei, J., Tzvetkov, M.V., Gal, V., Sachse-Seeboth, C., Sehrt, D., Hjelmborg, J.B., Hofmann, U., Schwab, M., Kerb, R., and Brockmöller, J. (2016). Low heritability in pharmacokinetics of talinolol: a pharmacogenetic twin study on the heritability of the pharmacokinetics of talinolol, a putative probe drug of MDR1 and other membrane transporters. *Genome Medicine* 8.

Maurer, T.S., Debartolo, D.B., Tess, D.A., and Scott, D.O. (2005). Relationship between exposure and nonspecific binding of thirty-three central nervous system drugs in mice. *Drug Metab Dispos* 33**,** 175-181.

Mutschler, E., Geisslinger, G., Kroemer, H.K., and Schäfer-Korting, M. (2001). *Mutschler Arzneimittelwirkungen: Lehrbuch der Pharmakologie und Toxikologie.* Stuttgart: Wissenschaftliche Verlagsgesellschaft.

Nakai, D., Nakagomi, R., Furuta, Y., Tokui, T., Abe, T., Ikeda, T., and Nishimura, K. (2001). Human liver-specific organic anion transporter, LST-1, mediates uptake of pravastatin by human hepatocytes. *J Pharmacol Exp Ther* 297**,** 861-867.

National Center for Biotechnology Information (2022a). *PubChem Compound Summary for CID 68770, Talinolol* [Online]. Available: https://pubchem.ncbi.nlm.nih.gov/compound/Talinolol [Accessed 2022-02-24].

National Center for Biotechnology Information (2022b). *PubChem Compound Summary for CID 5484731, Morphine-3-glucuronide* [Online]. Available: https://pubchem.ncbi.nlm.nih.gov/compound/Morphine-3-glucuronide [Accessed 2022-02-23].

Nehlig, A. (2018). Interindividual Differences in Caffeine Metabolism and Factors Driving Caffeine Consumption. *Pharmacol Rev* 70**,** 384-411.

Nguyen, H.Q., Kimoto, E., Callegari, E., and Obach, R.S. (2016). Mechanistic Modeling to Predict Midazolam Metabolite Exposure from In Vitro Data. *Drug Metab Dispos* 44**,** 781-791.

Obach, R.S., Lombardo, F., and Waters, N.J. (2008). Trend analysis of a database of intravenous pharmacokinetic parameters in humans for 670 drug compounds. *Drug Metab Dispos* 36**,** 1385-1405.

Oertel, R., Richter, K., Trausch, B., Berndt, A., Gramatté, T., and Kirch, W. (1994). Elucidation of the structure of talinolol metabolites in man determination of talinolol and hydroxylated talinolol metabolites in urine and analysis of talinolol in serum. *Journal of Chromatography B: Biomedical Sciences and Applications* 660**,** 353-363.

Ogura, K., Choudhuri, S., and Klaassen, C.D. (2000). Full-length cDNA cloning and genomic organization of the mouse liver-specific organic anion transporter-1 (lst-1). *Biochem Biophys Res Commun* 272**,** 563-570.

Perloff, M.D., Von Moltke, L.L., Court, M.H., Kotegawa, T., Shader, R.I., and Greenblatt, D.J. (200). Midazolam and Triazolam Biotransformation in Mouse and Human Liver Midrocomes: Relative Contribution of CYP3A and CYP2C Isoforms. *The Journal of pharmacology and experimental therapeutics* 292**,** 618-628.

Poulin, P., and Theil, F.P. (2002). Prediction of pharmacokinetics prior to in vivo studies. 1. Mechanism-based prediction of volume of distribution. *J Pharm Sci* 91**,** 129-156.

Puttegowda, V.D., Karki, R., Goli, D., Jha, S.K., and Mudagal, M.P. (2016). Formulation and Pharmacokinetic Evaluation of Microcapsules Containing Pravastatin Sodium Using Rats. *Scientifica* 2016**,** 7623193.

R Core Team (2020). "A language and environment for statistical computing". (Vienna, Austria: R Foundation for Statistical Computing).

Renaud, H.J., Cui, J.Y., Khan, M., and Klaassen, C.D. (2011). Tissue distribution and gender-divergent expression of 78 cytochrome P450 mRNAs in mice. *Toxicol Sci* 124**,** 261-277.

Schenk, A., Ghallab, A., Hofmann, U., Hassan, R., Schwarz, M., Schuppert, A., Schwen, L.O., Braeuning, A., Teutonico, D., Hengstler, J.G., and Kuepfer, L. (2017). Physiologically-based modelling in mice suggests an aggravated loss of clearance capacity after toxic liver damage. *Scientific Reports* 7.

Schmitt, W. (2008). General approach for the calculation of tissue to plasma partition coefficients. *Toxicology in Vitro* 22**,** 457-467.

Schwarz, U.I., Dresser, G.K., Oertel, R., and Kim, R.B. (2001). Talinolol-verapramil interaction is not solely due to P-glycoprotein inhibition. *Clin. Pharmacol. Ther.* 69**,** PIII-86.

Seo, K.A., Bae, S.K., Choi, Y.K., Choi, C.S., Liu, K.H., and Shin, J.G. (2010). Metabolism of 1'- and 4-hydroxymidazolam by glucuronide conjugation is largely mediated by UDP-glucuronosyltransferases 1A4, 2B4, and 2B7. *Drug Metab Dispos* 38**,** 2007-2013.

Serajuddin, A.T., Ranadive, S.A., and Mahoney, E.M. (1991). Relative lipophilicities, solubilities, and structure-pharmacological considerations of 3-hydroxy-3-methylglutaryl-coenzyme A (HMG-CoA) reductase inhibitors pravastatin, lovastatin, mevastatin, and simvastatin. *J Pharm Sci* 80**,** 830-834.

Terhaag, B., Gramatté, T., Richter, K., Voss, J., and Feller, K. (1989). The biliary elimination of the selective beta-receptor blocking drug talinolol in man. *Int J Clin Pharmacol Ther Toxicol* 27**,** 170-172.

Thiel, C., Schneckener, S., Krauss, M., Ghallab, A., Hofmann, U., Kanacher, T., Zellmer, S., Gebhardt, R., Hengstler, J.G., and Kuepfer, L. (2015). A Systematic Evaluation of the Use of Physiologically Based Pharmacokinetic Modeling for Cross-Species Extrapolation. *Journal of Pharmaceutical Sciences* 104**,** 191-206.

Trausch, B., Oertel, R., Richter, K., and Gramatte, T. (1995). Disposition and bioavailability of the beta 1-adrenoceptor antagonist talinolol in man. *Biopharm Drug Dispos* 16**,** 403-414.

Tubic, M., Wagner, D., Spahn-Langguth, H., Bolger, M.B., and Langguth, P. (2006). In silico modeling of non-linear drug absorption for the P-gp substrate talinolol and of consequences for the resulting pharmacodynamic effect. *Pharm Res* 23**,** 1712-1720.

Tzvetkov, M.V., Dos Santos Pereira, J.N., Meineke, I., Saadatmand, A.R., Stingl, J.C., and Brockmöller, J. (2013). Morphine is a substrate of the organic cation transporter OCT1 and polymorphisms in OCT1 gene affect morphine pharmacokinetics after codeine administration. *Biochemical Pharmacology* 86**,** 666-678.

Van De Wetering, K., Zelcer, N., Kuil, A., Feddema, W., Hillebrand, M., Vlaming, M.L.H., Schinkel, A.H., Beijnen, J.H., and Borst, P. (2007). Multidrug Resistance Proteins 2 and 3 Provide Alternative Routes for Hepatic Excretion of Morphine-Glucuronides. *Molecular Pharmacology* 72**,** 387-394.

Van Waterschoot, R.a.B., Van Herwaarden, A.E., Lagas, J.S., Sparidans, R.W., Wagenaar, E., Van Der Kruijssen, C.M.M., Goldstein, J.A., Zeldin, D.C., Beijnen, J.H., and Schinkel, A.H. (2007). Midazolam Metabolism in Cytochrome P450 3A Knockout Mice Can Be Attributed to Up-Regulated CYP2C Enzymes. *Molecular Pharmacology* 73**,** 1029-1036.

Varkhede, N., Patel, N., Chang, W., Ruterbories, K., and Forrest, M.L. (2018). A Semi-Physiologically Based Pharmacokinetic Model Describing the Altered Metabolism of Midazolam Due to Inflammation in Mice. *Pharm Res* 35**,** 162.

Vossen, M., Sevestre, M., Niederalt, C., Jang, I.J., Willmann, S., and Edginton, A.N. (2007). Dynamically simulating the interaction of midazolam and the CYP3A4 inhibitor itraconazole using individual coupled whole-body physiologically-based pharmacokinetic (WB-PBPK) models. *Theor Biol Med Model* 4**,** 13.

Vree, T.B., and Verwey-Van Wissen, C.P. Pharmacokinetics and metabolism of codeine in humans.

Wishart, D.S., Feunang, Y.D., Guo, A.C., Lo, E.J., Marcu, A., Grant, J.R., Sajed, T., Johnson, D., Li, C., Sayeeda, Z., Assempour, N., Iynkkaran, I., Liu, Y., Maciejewski, A., Gale, N., Wilson, A., Chin, L., Cummings, R., Le, D., Pon, A., Knox, C., and Wilson, M. (2017). DrugBank 5.0: a major update to the DrugBank database for 2018. *Nucleic Acids Research* 46**,** D1074-D1082.

Yu, A.M., and Haining, R.L. (2006). Expression, purification, and characterization of mouse CYP2d22. *Drug Metab Dispos* 34**,** 1167-1174.

Zaher, H., Meyer Zu Schwabedissen, H.E., Tirona, R.G., Cox, M.L., Obert, L.A., Agrawal, N., Palandra, J., Stock, J.L., Kim, R.B., and Ware, J.A. (2008). Targeted disruption of murine organic anion-transporting polypeptide 1b2 (Oatp1b2/Slco1b2) significantly alters disposition of prototypical drug substrates pravastatin and rifampin. *Mol Pharmacol* 74**,** 320-329.

Zamek-Gliszczynski, M.J., Hoffmaster, K.A., Nezasa, K., Tallman, M.N., and Brouwer, K.L. (2006). Integration of hepatic drug transporters and phase II metabolizing enzymes: mechanisms of hepatic excretion of sulfate, glucuronide, and glutathione metabolites. *Eur J Pharm Sci* 27**,** 447-486.

Zanger, U.M., and Schwab, M. (2013). Cytochrome P450 enzymes in drug metabolism: Regulation of gene expression, enzyme activities, and impact of genetic variation. *Pharmacology & Therapeutics* 138**,** 103-141.

Zelcer, N., Van De Wetering, K., Hillebrand, M., Sarton, E., Kuil, A., Wielinga, P.R., Tephly, T., Dahan, A., Beijnen, J.H., and Borst, P. (2005). Mice lacking multidrug resistance protein 3 show altered morphine

pharmacokinetics and morphine-6-glucuronide antinociception. *Proceedings of the National Academy of Sciences* 102, 7274-7279.
